# Effect of moderate-intensity statin with ezetimibe combination vs. high-intensity statin therapy according to sex in patients with atherosclerosis

**DOI:** 10.1038/s41598-023-47505-x

**Published:** 2023-11-17

**Authors:** Byung Gyu Kim, Seung-Jun Lee, Yong-Joon Lee, Seng Chan You, Soon Jun Hong, Kyeong Ho Yun, Bum-Kee Hong, Jung Ho Heo, Seung-Woon Rha, Sung-Jin Hong, Chul-Min Ahn, Byeong-Keuk Kim, Young-Guk Ko, Donghoon Choi, Myeong-Ki Hong, Yangsoo Jang, Yun-Hyeong Cho, Jung-Sun Kim

**Affiliations:** 1 Sanggye Paik Hospital, Inje University College of Medicine, Seoul, Korea; 2 Division of Cardiology, Severance Hospital, Yonsei University College of Medicine, Yonsei-ro 50-1, Seodaemun-gu, Seoul, 03722 Korea; 3 Department of Biomedical Systems Informatics, Yonsei University College of Medicine, Seoul, Korea; 4 Korea University College of Medicine, Seoul, Korea; 5 Wonkwang University Hospital, Iksan, Korea; 6 Gangnam Severance Hospital, Yonsei University College of Medicine, Seoul, Korea; 7 Kosin University College of Medicine, Busan, Korea; 8 Korea University Guro Hospital, Seoul, Korea; 9 CHA Bundang Medical Center, CHA University College of Medicine, Seongnam, Korea; 10 Division of Cardiology, Myongji Hospital, Hwasu-ro 14-55, Deogyang-gu, Goyang, 10475 Gyeonggi-do Korea

**Keywords:** Cardiology, Cardiovascular diseases, Outcomes research

## Abstract

We aimed to evaluate sex differences in the effects of moderate-intensity statin with ezetimibe combination therapy (rosuvastatin 10 mg plus ezetimibe) versus high-intensity statin (rosuvastatin 20 mg) monotherapy in patients with atherosclerotic cardiovascular disease (ASCVD). This was a sex-specific subgroup analysis of the RACING trial that evaluated the interaction between sex and treatment strategies for the primary outcome (composite of cardiovascular death, major cardiovascular events, or nonfatal stroke at 3 years). Of 3780 patients in the RACING trial, 954 (25.2%) were women. Regardless of sex, the effect of moderate-intensity statin with ezetimibe combination therapy on primary outcome compared with high-intensity statin monotherapy was similar (hazard ratio [HR] 0.98 [0.63–1.52] in women; HR 0.90 [0.71–1.14] in men). The rate of discontinuation or dose reduction of study drugs due to intolerance was lower in the ezetimibe combination group than in the high-intensity statin monotherapy group in both women (4.5% vs. 8.6%, P = 0.014) and men (4.8% vs. 8.0%, P < 0.001). LDL cholesterol levels of < 70 mg/dL at 1, 2, and 3 years were more frequently achieved in the ezetimibe combination group than in the high-intensity statin monotherapy group (all P < 0.001) in both sexes. There were no significant interactions between sex and treatment groups regarding the primary outcome, discontinuation, or dose reduction of study drugs, or the proportion of achievement of LDL cholesterol levels < 70 mg/dL. The effect of ezetimibe combination therapy for the 3-year composite outcomes was not different in both men and women. The benefits of ezetimibe combination therapy on LDL cholesterol lowering and drug tolerance were similarly observed regardless of sex.

Trial registration: https://clinicaltrials.gov; Unique identifier: NCT03044665.

## Introduction

Recent guidelines recommend the use of high-intensity statin therapy in patients with established atherosclerotic cardiovascular disease (ASCVD). However, it did not make a distinction stratified by sex^1–3^, because a large meta-analysis of 22 trials of statin vs. control and five trials of more- vs. less-intensive statin therapy demonstrated that the proportional reductions per mmol/L reduction in low-density lipoprotein (LDL) cholesterol in major clinical ASCVD were similar between women and men after adjustment for non-sex profiles^4^. Despite the similar efficacy of intensive statin therapy in both sexes, numerous studies have consistently reported that women are less likely to use high-intensity statins than men for the secondary prevention of ASCVD^5–8^. Therefore, the cause of the observed sex disparity in statin use remains unclear^9^. Some data has demonstrated that women are more likely to be non-adherent to statins than men, which might be associated with a higher rate of side effects^10,11^. Rather than increasing the dose or intensity of statins in women intolerant to statins, combination therapy with statins and ezetimibe may be an alternative strategy^12^. The addition of ezetimibe to statins did not appear to increase the incidence of elevated serum creatine kinase levels beyond that observed with statin treatment alone^13^. However, the sex-specific effect of ezetimibe and moderate-intensity combination therapy versus high-intensity statin therapy alone in reducing adverse events remains uncertain. In the result of Randomized Comparison of Efficacy and Safety of Lipid-lowering with Statin Monotherapy Versus Statin/ezetimibe Combination for High-risk Cardiovascular Disease (RACING) study, moderate-intensity statin with ezetimibe combination therapy was found to be comparable with high-intensity statin monotherapy in terms of the 3-year composite cardiovascular outcomes in patients with ASCVD^14^. Whether these effects are sex dependent remains unknown. Accordingly, we evaluated the sex-dependent effect of moderate-intensity statin with ezetimibe combination (rosuvastatin 10 mg plus ezetimibe) therapy versus high-intensity statin (rosuvastatin 20 mg) monotherapy on 3-year composite outcomes in patients with ASCVD as a pre-specified analysis of the RACING trial.

## Methods

Data regarding this article will be shared by the corresponding author upon reasonable request.

### Trial design and population

**Figure 1:**
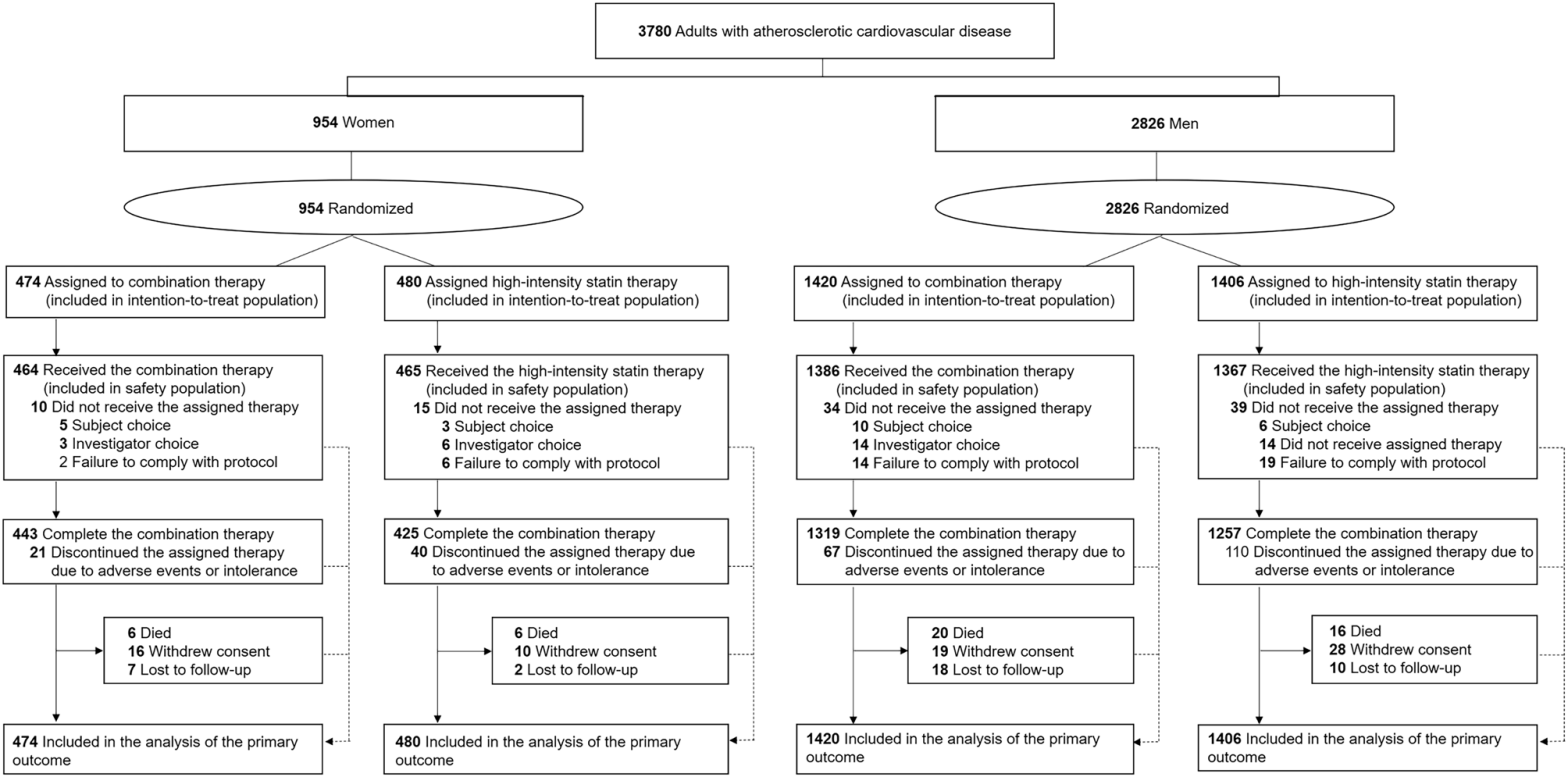
Study flow of participants.

This study was a pre-specified subgroup analysis of the RACING trial. The RACING trial (https://clinicaltrials.gov; Unique identifier: NCT03044665, registration date: 07/02/2017) was a Korean multicenter randomized trial investigating the efficacy and safety of moderate-intensity statin with ezetimibe combination therapy (ezetimibe combination therapy) versus high-intensity statin monotherapy in patients with established ASCVD. Detailed explanations, including the study design, rationale, and inclusion and exclusion criteria, have been described elsewhere^14^. The trial was approved by the institutional review board of each center (Yonsei University Health System, Institutional Review Board, 4-2016-1025) and was performed in accordance with the principles of the Declaration of Helsinki. All the participants provided written informed consent. The results of study were reported in adherence to the CONSORT reporting guidelines. For the present investigation, the patients were divided into two groups according to sex (Fig. 1).

### Study procedures

The RACING trial randomly assigned patients in a 1:1 fashion to receive either ezetimibe combination therapy (rosuvastatin 10 mg with ezetimibe 10 mg) or rosuvastatin 20 mg monotherapy. The initial doses of the study drugs were strongly recommended for maintenance throughout the study period. However, considering the patients’ tolerance, compliance, and various clinical situations, the discontinuation or alteration of doses in both treatment groups was decided at the physicians’ discretion, and a detailed report of reasons was required. Clinical follow-up for assessment of muscle-related symptoms, medication use, and the occurrence of study outcomes was performed at 2 and 6 months, and at 1, 2, and 3 years of follow-up. Patients’ lipid profiles (total cholesterol, LDL cholesterol, high-density lipoprotein cholesterol, and triglyceride levels) were examined serially at 1, 2, and 3 years.

### Study outcomes

The primary outcome included cardiovascular death, major cardiovascular events, or nonfatal stroke within 3 years^14^. Major cardiovascular events included coronary or peripheral artery revascularization or hospitalization for cardiovascular events^14^. Cardiovascular death was defined as death from myocardial infarction (MI), heart failure, stroke, cardiovascular procedures, cardiovascular hemorrhage, sudden cardiac death, or any death in which a cardiovascular cause could not be excluded as adjudicated by a clinical endpoints committee^15^. MI was defined as a creatine kinase MB fraction above the upper normal limit or a troponin T or troponin I level greater than the 99th percentile of the upper reference limit, with concomitant ischemic symptoms or electrocardiographic findings, or abnormal findings on imaging studies indicative of ischemia^15^. Coronary or peripheral revascularization comprises both endovascular and surgical revascularization of the coronary, carotid, or lower extremity arteries^14,16^. Hospitalization for cardiovascular events was defined as a hospitalization for ischemic heart disease, heart failure, or peripheral artery disease^15,17,18^. Nonfatal stroke was defined as occurrence of a focal neurological deficit more than 24 h or the presence of acute infarction confirmed by brain imaging studies^19^.

The secondary outcomes were clinical efficacy and safety. Efficacy outcomes were proportion of patients whose LDL cholesterol levels < 70 mg/dL at 1, 2, and 3 years; composite of all-cause death, major cardiovascular events, or nonfatal stroke; decrease in the concentration of LDL cholesterol, that is, the percentage reduction of LDL cholesterol from baseline to follow-up; and any individual component of the primary outcome^14^. As a post-hoc analysis, the proportion of patients who achieved a LDL cholesterol level < 55 mg/dL was also analyzed, since the latest 2019 European Society of Cardiology/European Atherosclerosis Society guidelines recommended the new LDL cholesterol target goal of < 55 mg/dL for secondary prevention in patients with ASCVD after the initiation of the RACING trial^3,14^. Safety outcomes included the discontinuation or dose reduction of the study drug due to intolerance and the occurrence of clinical adverse events including new-onset diabetes, muscle-, hepatic-, or gallbladder-related adverse events or cancer diagnosis^14^. Given that heart failure events are theoretically considered unaffected by lipid lowering therapies, an additional analysis was performed by excluding hospitalization for heart failure from the primary outcome and restricting the outcomes to atherosclerotic cardiovascular events.

### Statistical analyses

Continuous variables were expressed as mean ± standard deviation or median (interquartile range), depending on their distribution, and categorical data as numbers (frequencies). Baseline and procedural characteristics among the groups were compared using Student’s *t*-test or Mann–Whitney *U* test for continuous variables and Chi-square or Fisher’s exact test for categorical variables. The primary and secondary efficacy outcomes were analyzed based on an intention-to-treat approach. For the analyses of secondary safety outcomes, the safety population was considered, excluding patients who did not receive the assigned therapy unless they stopped or reduced dose due to intolerance. Sensitivity analyses were conducted in the intention-to-treat population regarding secondary safety outcomes. Log-rank and Kaplan–Meier tests were used to compare adverse event rates between the treatment groups. Hazard ratios (HRs) for clinical outcomes according to sex were assessed using a Cox regression model and are shown with 95% confidence intervals (CI). The models were adjusted for variables displaying baseline differences or known biological confounders, including age, body mass index, prior MI, prior percutaneous coronary artery intervention, hypertension, chronic kidney disease, current smoking status, and baseline low-density lipoprotein cholesterol level. The treatment effect heterogeneity among the subgroups was assessed using interaction terms in the Cox proportional hazards or logistic regression models, as appropriate. All tests were two sided. Statistical significance was set at P < 0.05. Statistical analyses were performed using the R Statistical Software (version 3.5.3; R Foundation for Statistical Computing, Vienna, Austria).

## Results

### Participant characteristics

Of the 3780 patients randomized in the RACING trial, 954 (25.2%) were women. The baseline characteristics and laboratory findings of the study population according to sex are summarized in Table 1. Women, compared with men, were older (mean 67 versus 63 years), had a lower body weight (mean 60 versus 71 kg), and body mass index (24.9 versus 25.1 kg/m^2^); had more frequent diagnoses of chronic kidney disease (13.3 versus 9.4%) and hypertension (70.9 versus 65.3%). However, women had a lower proportion of prior MI (26.3 versus 48.3%), prior percutaneous coronary intervention (55.8 versus 69.5%), and current smoking (3.0 versus 21.5%). Women were more likely not to take lipid-lowering agents (17.3 versus 11.8%), had a higher serum LDL cholesterol level (mean 89.9 versus 83.5 mg/dL), and had a lower proportion of LDL cholesterol < 70 mg/dL (27.3 versus 35.4%). A comparison of baseline characteristics stratified by sex and treatment assignment is presented in Table S1. The baseline characteristics were well balanced between the study groups for both female and male patients.

**Table 1:** Baseline characteristics according to sex.

Characteristics	Women (n = 954)	Men (n = 2826)	P value
Age, y	67.4 ± 8.4	62.6 ± 9.7	< 0.001
Weight, kg	59.7 ± 8.9	71.2 ± 10.3	< 0.001
Body mass index, kg/m^2^	24.9 ± 3.4	25.1 ± 3.0	0.044
Previous myocardial infarction	251 (26.3)	1238 (43.8)	< 0.001
Previous percutaneous coronary intervention	532 (55.8)	1965 (69.5)	< 0.001
Previous coronary bypass graft surgery	54 (5.7)	193 (6.8)	0.235
Acute coronary syndrome	11 (1.2)	36 (1.3)	0.903
Previous ischemic stroke	56 (5.9)	157 (5.6)	0.777
Chronic kidney disease*	127 (13.3)	265 (9.4)	0.001
End-stage kidney disease on dialysis	10 (1.0)	19 (0.7)	0.349
Peripheral artery disease	37 (3.9)	98 (3.5)	0.624
Hypertension	676 (70.9)	1844 (65.3)	0.002
Diabetes	338 (35.4)	1060 (37.5)	0.266
Diabetes with insulin	34 (3.6)	86 (3.0)	0.492
Current smoker	29 (3.0)	609 (21.5)	< 0.001
Medication for dyslipidemia before randomization			< 0.001
High-intensity statin	72 (7.5)	244 (8.6)	
High-intensity statin with ezetimibe	36 (3.7)	112 (4.0)	
Moderate-intensity statin	356 (37.3)	1010 (35.7)	
Moderate-intensity statin with ezetimibe	165 (17.3)	334 (11.8)	
Low-intensity statin	1 (0.1)	10 (0.4)	
None	165 (17.3)	334 (11.8)	
Serum LDL cholesterol concentration, mg/dL	89.9 ± 32.7	83.5 ± 30.7	< 0.001
Number of patients with LDL cholesterol concentration < 70 mg/dL	260 (27.3)	999 (35.4)	< 0.001

Data are presented as mean ± SD, median (interquartile range), or number (%). *LDL* low-density lipoprotein cholesterol.

*Chronic kidney disease was defined as an estimated glomerular filtration rate of less than 60 mL per min per 1.73 m^2^ of body surface area.

### Clinical efficacy and safety according to sex

Patients were followed-up for a median of 3.0 years (interquartile range, 3.0–3.0 years). The 3-year clinical outcomes according to sex are shown in Fig. S1. The primary outcome (78 women [8.2%] vs. 280 men [9.9%]; HR 0.82; 95% CI 0.64–1.06; P = 0.127) and secondary outcomes (83 women [8.7%] vs. 300 men [10.6%]; HR 0.82; 95% CI 0.64–1.04; P = 0.103) tended to occur less frequently in women than in men; however, the difference was not statistically significant. After multivariate adjustment, the incidence of secondary outcomes was significantly lower in women than in men (adjusted HR 0.75; 95% CI 0.58–0.97; P = 0.030). There was no difference between the sexes in individual clinical outcomes. The rate of secondary safety outcomes did not differ between sexes (Table S2).

### Clinical efficacy and safety according to sex and treatment assignment

**Figure 2:**
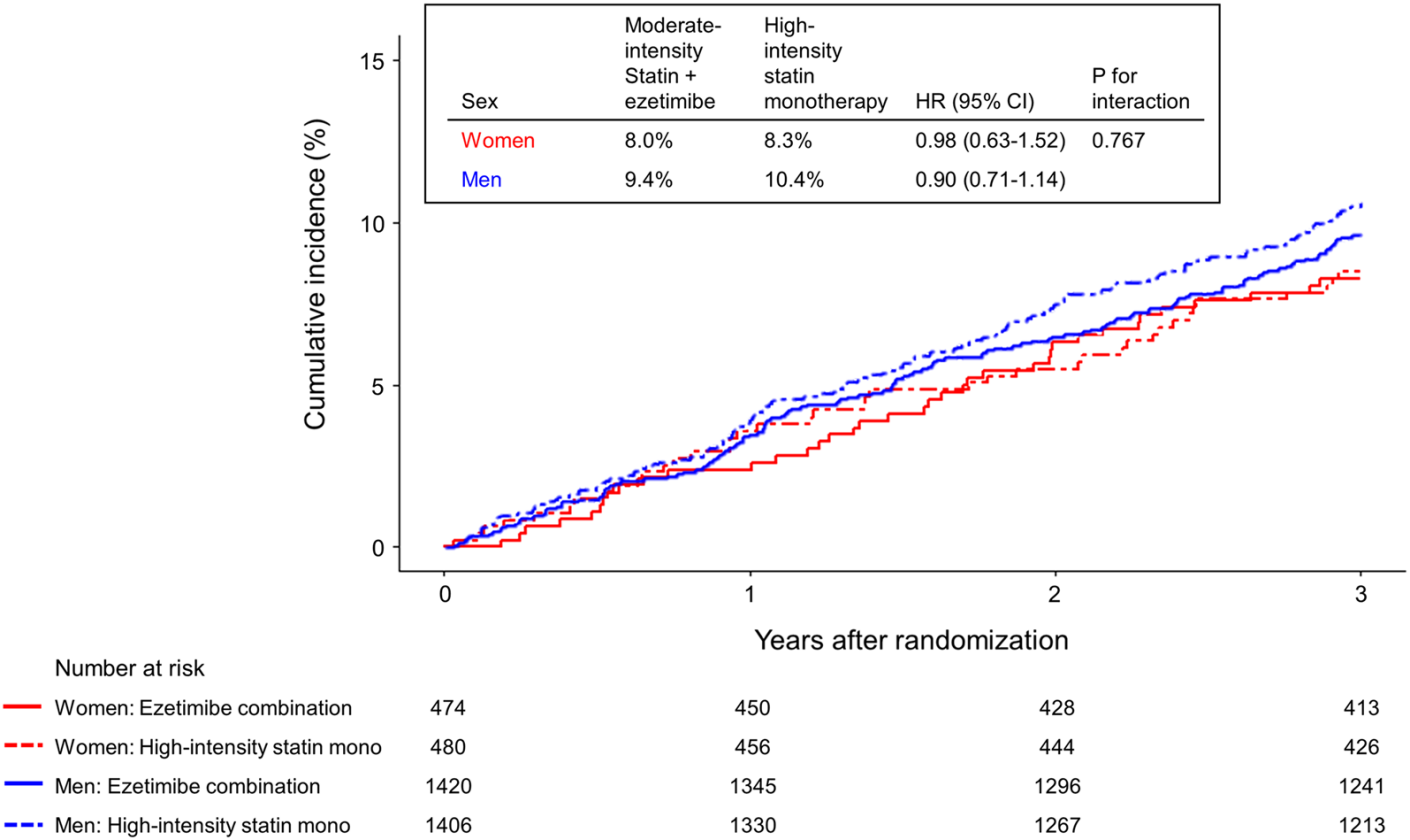
Time-to-event curves of primary outcome in women and men. Kaplan–Meier curves for the primary outcome according to sex and treatment assignment. *CI* confidence interval, *HR* hazard ratio.

Compared with rosuvastatin 20 mg monotherapy, the effect of ezetimibe combination therapy on primary outcome did not differ between women (HR 0.98; 95% CI 0.63–1.52; P = 0.911) and men (HR 0.90; 95% CI 0.71–1.14; P = 0.394) (Table 2 and Fig. 2). The rates of developing other secondary outcomes and individual clinical outcomes did not differ between the treatment groups in both women and men. These findings were consistent after multivariate adjustment (Table S3). There were no interactions between sex and lipid-lowering strategies for primary, secondary, or individual clinical outcomes (all P for interaction > 0.05).

**Table 2:** Primary and secondary efficacy outcomes according to sex and therapy strategy.

	Women (n = 954)				Men (n = 2826)				P for interaction*
	Ezetimibe combination therapy (n = 474)	High-intensity statin monotherapy (n = 480)	HR (95% CI)	P value	Ezetimibe combination therapy (n = 1420)	High-intensity statin monotherapy (n = 1406)	HR (95% CI)	P value	
Primary outcome									
Composite of cardiovascular death, major cardiovascular events, or nonfatal stroke	38 (8.0)	40 (8.3)	0.98 (0.63–1.52)	0.911	134 (9.4)	146 (10.4)	0.90 (0.71–1.14)	0.394	0.767
Secondary outcome									
Composite of all-cause death, major cardiovascular events, or nonfatal stroke	41 (8.6)	42 (8.8)	1.00 (0.65–1.54)	0.992	145 (10.2)	155 (11.0)	0.92 (0.73–1.15)	0.473	0.734
Individual clinical outcome									
Cardiovascular death	2 (0.4)	2 (0.4)	1.03 (0.15–7.33)	0.974	6 (0.4)	4 (0.3)	1.49 (0.42–5.27)	0.539	0.759
All-cause death	6 (1.3)	6 (1.2)	1.04 (0.33–3.21)	0.951	20 (1.4)	16 (1.1)	1.24 (0.64–2.39)	0.521	0.785
Major cardiovascular events	35 (7.4)	37 (7.7)	0.97 (0.61–1.54)	0.894	118 (8.3)	130 (9.2)	0.89 (0.70–1.15)	0.381	0.767
Coronary artery revascularization	18 (3.8)	18 (3.8)	1.03 (0.53–1.98)	0.935	73 (5.1)	71 (5.0)	1.02 (0.73–1.41)	0.912	0.978
Percutaneous coronary intervention	17 (3.6)	18 (3.8)			70 (5.0)	71 (5.0)			
Coronary artery bypass surgery	1 (0.2)	0			3 (0.2)	0			
Peripheral artery revascularization	0	0	–	–	8 (0.6)	7 (0.5)	1.14 (0.41–3.13)	0.807	–
Hospitalization for ischemic heart disease	32 (6.8)	30 (6.2)	1.10 (0.67–1.81)	0.712	110 (7.7)	120 (8.5)	0.90 (0.70–1.17)	0.438	0.493
Stable angina or unstable angina	27 (5.7)	27 (5.6)			93 (6.5)	106 (7.5)			
Acute myocardial infarction	5 (1.1)	3 (0.6)			17 (1.2)	14 (1.0)			
Hospitalization for heart failure	6 (1.3)	7 (1.5)	0.88 (0.30–2.63)	0.823	8 (0.6)	12 (0.9)	0.66 (0.27–1.62)	0.364	0.689
Hospitalization for peripheral artery disease	0	0	–	–	8 (0.6)	7 (0.5)	1.14 (0.41–3.13)	0.807	–
Nonfatal stroke	2 (0.4)	1 (0.2)	2.07 (0.19–22.83)	0.552	13 (0.9)	13 (0.9)	0.99 (0.46–2.14)	0.981	0.567
Ischemic stroke	1 (0.2)	1 (0.2)			10 (0.7)	10 (0.7)			
Hemorrhagic stroke	1 (0.2)	0			3 (0.2)	3 (0.2)			

Data are presented as number (% of the cumulative rates at 3 years according to Kaplan–Meier event rates). *CI* confidence interval, *HR* hazard ratio.

*P-value for interaction between sex and therapy.

**Figure 3:**
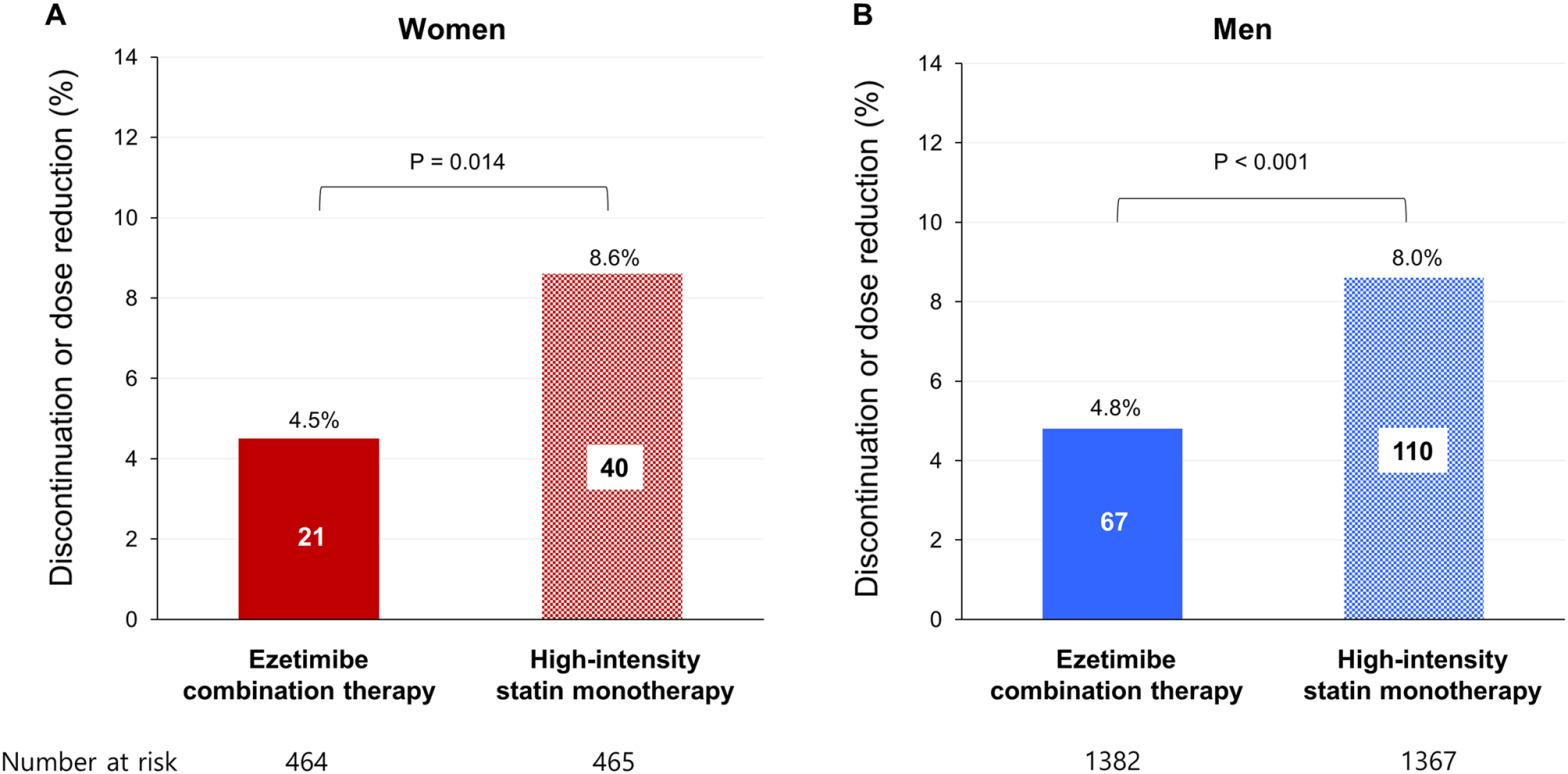
Drug discontinuation or dose reduction due to intolerance in women and men. Rates of discontinuation or dose reduction of the study drug due to intolerance in women (**A**) and men (**B**) in the safety population.

The rate of discontinuation or dose reduction of the study drug due to intolerance was lower in the ezetimibe combination therapy than in the rosuvastatin 20 mg monotherapy group in both women (4.5 vs. 8.6%; P = 0.014) and men (4.8 vs. 8.0%; P < 0.001) (Table 3 and Fig. 3). Other secondary safety outcomes related to the study drugs are shown in Table 3, which did not differ between the two treatment groups, regardless of sex.

**Table 3:** Secondary safety outcomes according to sex and therapy strategy in the safety population.

	Women (n = 929)			Men (n = 2749)			P for interaction^†^
	Ezetimibe combination therapy (n = 464)	High-intensity statin monotherapy (n = 465)	P value	Ezetimibe combination therapy (n = 1382)	High-intensity statin monotherapy (n = 1367)	P value	
Discontinuation or dose reduction of the study drug due to intolerance	21 (4.5)	40 (8.6)	0.014	67 (4.8)	110 (8.0)	< 0.001	0.651
Patients’ reported symptoms							
Dizziness or general weakness	2 (0.4)	6 (1.3)		8 (0.6)	15 (1.1)		
Chest discomfort or headache	2 (0.4)	2 (0.4)		5 (0.4)	10 (0.7)		
Gastrointestinal symptom	1 (0.2)	1 (0.2)		3 (0.2)	8 (0.6)		
Urticaria or itching sensation	2 (0.4)	4 (0.9)		4 (0.3)	3 (0.2)		
Myalgia	2 (0.4)	6 (1.3)		5 (0.4)	16 (1.2)		
Other	4 (0.9)	2 (0.4)		1 (0.1)	1 (0.1)		
Physicians’ discretion							
Liver enzyme elevation	4 (0.9)	6 (1.3)		11 (0.8)	26 (1.9)		
Creatine kinase elevation	2 (0.4)	10 (2.2)		23 (1.7)	23 (1.7)		
Fasting glucose level elevation	1 (0.2)	2 (0.4)		4 (0.3)	4 (0.3)		
Other	1 (0.2)	1 (0.2)		3 (0.2)	4 (0.3)		
New-onset diabetes	58 (12.5)	52 (11.2)	0.535	146 (10.6)	143 (10.5)	0.929	0.629
New-onset diabetes with initiation of anti-diabetic medication	19 (4.1)	26 (5.6)	0.290	76 (5.5)	81 (5.9)	0.630	0.479
Muscle-related adverse events	3 (0.6)	9 (1.9)	0.098	18 (1.3)	25 (1.8)	0.268	0.301
Myalgia	3 (0.6)	7 (1.5)	0.218	14 (1.0)	22 (1.6)	0.173	0.619
Myopathy	0	0	–	2 (0.1)	4 (0.3)	0.416	–
Myonecrosis*	0	3 (0.6)	0.994	11 (0.8)	10 (0.7)	0.846	0.985
Mild	0	1 (0.2)		8 (0.6)	8 (0.6)		
Moderate	0	1 (0.2)		2 (0.2)	2 (0.2)		
Severe including rhabdomyolysis	0	1 (0.2)		1 (0.1)	0		
Gallbladder-related adverse events	1 (0.2)	1 (0.2)	0.999	11 (0.8)	6 (0.4)	0.239	0.692
Major bleeding	2 (0.4)	3 (0.6)	0.658	13 (0.9)	10 (0.7)	0.548	0.513
Cancer diagnosis	5 (1.1)	5 (1.1)	0.997	32 (2.3)	21 (1.5)	0.140	0.550
New-onset neurocognitive disorder	1 (0.2)	1 (0.2)	0.999	3 (0.2)	1 (0.1)	0.346	0.552
Cataract surgery	4 (0.9)	6 (1.3)	0.530	15 (1.1)	15 (1.1)	0.976	0.595

Data are presented as number (%).

*Severity of myonecrosis was classified by an elevation of creatine kinase level compared with either the baseline level or the upper limit of normal (ULN); mild, 3–10 times ULN; moderate, 10–50 times ULN; severe, > 50 times ULN.

^†^P-value for interaction between sex and therapy.

### Change in lipids according to sex and treatment assignment

The serial changes in LDL cholesterol levels by sex and treatment group during the study period are presented in Table 4 and Fig. S2. Regardless of sex, median LDL cholesterol levels were consistently lower in the ezetimibe combination therapy group than in the rosuvastatin 20 mg monotherapy group at 1, 2, and 3 years of follow-up (all P < 0.001). The achievement rate of LDL cholesterol levels < 70 mg/dL was significantly higher in the ezetimibe combination therapy group than in the rosuvastatin 20 mg monotherapy group in both sexes (all P < 0.001). There was no interaction between sex and treatment groups in the proportion of patients with LDL-cholesterol levels < 70 mg/dL. As a post-hoc analysis, the achievement rate of LDL cholesterol levels < 55 mg/dL at 1, 2, and 3 years was also evaluated, and was consistently higher in the ezetimibe combination therapy group than in the rosuvastatin 20 mg monotherapy group in both sexes (all P < 0.001) (Table S4). Serial changes in the other lipid profiles are summarized in Table S5. Total cholesterol and triglyceride levels were lower in ezetimibe combination therapy than rosuvastatin 20 mg monotherapy group, whereas high-density lipoprotein cholesterol levels were not different between two treatment groups among both female and male patients.

**Table 4:** Serial LDL cholesterol levels according to sex and therapy strategy.

	Women (n = 954)			Men (n = 2826)			P for interaction*
	Ezetimibe combination therapy	High-intensity statin monotherapy	P value	Ezetimibe combination therapy	High-intensity statin monotherapy	P value	
1 year							
Number of patients	428	434		1247	1239		
LDL cholesterol level, mg/dL	61 (50–74)	71 (58–86)	< 0.001	57 (46–70)	65 (54–78)	< 0.001	
Number of patients with LDL cholesterol levels < 70 mg/dL (%)	282 (65.9)	201 (46.3)	< 0.001	935 (75.0)	722 (58.3)	< 0.001	0.797
2 years							
Number of patients	391	389		1167	1148		
LDL cholesterol level, mg/dL	60 (48–74)	67 (55–83)	< 0.001	56 (44–68)	64 (52–78)	< 0.001	
Number of patients with LDL cholesterol levels < 70 mg/dL (%)	274 (70.1)	208 (53.5)	< 0.001	894 (76.6)	716 (62.3)	< 0.001	0.881
3 years							
Number of patients	339	335		1010	979		
LDL cholesterol level, mg/dL	58 (48–72)	68 (57–81)	< 0.001	58 (46–70)	65 (53–79)	< 0.001	
Number of patients with LDL cholesterol levels < 70 mg/dL (%)	239 (70.5)	173 (51.6)	< 0.001	739 (73.2)	585 (59.8)	< 0.001	0.294

Data are presented as medians (interquartile ranges) or numbers (%). *DM* diabetes mellitus, *LDL* low-density lipoprotein cholesterol.

*P-value for the interaction between sex and therapy.

### Additional analysis

As a post-hoc analysis, the outcomes of atherosclerotic cardiovascular events were examined only after excluding hospitalization for heart failure. The occurrence of atherosclerotic cardiovascular events (a composite of cardiovascular death, coronary or peripheral revascularization, hospitalization for ischemic heart disease or peripheral artery disease, and non-fatal stroke) was not different between the ezetimibe combination therapy and rosuvastatin 20 mg monotherapy groups among women (7.4 vs. 6.9%, P = 0.728) and men (8.9 vs. 9.7%, P = 0.454), and there were no significant interaction (P for interaction 0.517) between sex and treatment strategies (Fig. S3).

## Discussion

In this pre-specified subgroup analysis from the RACING trial, we found that (i) despite substantial differences in baseline profiles between sexes, the adjusted risk of primary outcome did not differ between sexes during the 3-year follow-up in patients with ASCVD; (ii) regardless of sex, the risks of 3-year cardiovascular composite outcomes did not differ between the ezetimibe combination therapy and rosuvastatin 20 mg monotherapy groups, and there was no interaction between sex and treatment strategies; (iii) the ezetimibe combination therapy group showed a lower rate of drug discontinuation or dose reduction due to intolerance in both sexes; and (iv) the LDL cholesterol levels at follow-up were lower and the proportion of LDL cholesterol levels < 70 mg/dL was higher in the ezetimibe combination than in the rosuvastatin 20 mg monotherapy group in both women and men.

Current guidelines recommend the use of high-intensity statins as a first-line therapy to lower LDL cholesterol levels in patients with ASCVD, and this recommendation applies equally to both women and men^1–3^. The results of the Cholesterol Treatment Trialists’ meta-analyses showed that the proportional reductions in cardiovascular events according to LDL cholesterol reduction by statins were similar regardless of sex^4^. Despite the robust benefits of statins, the use of high-intensity statins is often limited because of non-adherence related to its side effects^20,21^. In particular, under-utilization of high-intensity statins and poor adherence in women rather than men are widely reported^5–8^. Furthermore, large proportion of patients on statin monotherapy fail to reach the target levels of LDL cholesterol, since the latest dyslipidemia guidelines set a dual goal of achieving LDL cholesterol levels less than 55 mg/dL and LDL cholesterol reduction of at least 50% from baseline in patients with ASCVD^3,22^. Non-statin therapies on top of statin therapy demonstrated an additional LDL cholesterol-lowering effect and better clinical outcomes^23,24^. In patients intolerant to high-intensity statins, a combination of ezetimibe and moderate-intensity statins is considered a reasonable alternative strategy^12,23^. However, data regarding the sex-specific beneficial effects and safety of non-statin combination therapies is limited. A dedicated analysis of sex-dependent effects on the benefit of adding ezetimibe to moderate-intensity statins versus the absence of ezetimibe (IMPROVI-IT; Improved Reduction of Outcomes: Vytorin Efficacy International Trial) demonstrated a comparable benefit for reducing primary outcome (a composite of cardiovascular death, major coronary events, or stroke) and LDL cholesterol levels in both women and men^25^. However, the effect of adding ezetimibe to moderate-intensity statin was compared with that of moderate-intensity statin, but not high-intensity statin^25^. Therefore, analyzing the sex-dependent effect on further head-to-head comparisons between moderate-intensity statin and ezetimibe combination therapy versus high-intensity statin monotherapy may provide valuable clinical implications considering the gap between current guidelines and real-world practice. In this dedicated and comprehensive analysis of the sex-dependent effect of the results of the RACING trial, moderate-intensity statin with ezetimibe combination therapy demonstrated a comparable 3-year composite outcome of cardiovascular death, major cardiovascular events, or non-fatal stroke in both women and men compared with high-intensity statin monotherapy, with a better LDL cholesterol lowering effect supporting the use of combination therapy, irrespective of sex.

In addition, our study showed that moderate-intensity statin plus ezetimibe combination therapy had better safety profiles than high-intensity statin monotherapy in both women and men. Although few studies have focused on sex differences in adverse events, including muscle-related symptoms, women are more likely to experience such side effects from statin therapy than men^10,11^, which may explain why women tend to be undertreated than men. As women generally have lower body weight or muscle mass than men and the statin dosages are not weight-based, high-intensity statin therapy may proportionally increase statin concentrations in women and increase the risk of stain-related side effects^26^. In our study, despite the differences of age, body weight, and baseline renal functions among women and men, the rate of drug intolerance, muscle-related adverse events or any other adverse events were not different between sexes, irrespective of treatment assignment, which coincides with the result of the sub-group analysis of IMPROVE-IT trial^25^. However, it should be taken into account that our data and IMPROVE-IT results have been obtained from randomized clinical trials that usually include selected patients in some way; thus, the rate of side effects might differ in real clinical practice. In analyses of several randomized clinical trials, the incidence of muscle symptoms without a significant increase in creatine kinase is similar between statin-treated and placebo-treated groups, whereas in real-world clinical practice, approximately 10% of patients stop taking statins due to their subjective complaints, most of which are muscle symptoms without an increase in creatine kinase, suggesting that they often stop taking statins due to the nocebo effect rather than the statin pharmacological effect^27,28^. Nevertheless, our findings that ezetimibe combination therapy, regardless of sex, has better efficacy and exhibits lower safety outcomes than high-intensity monotherapy, may help ameliorate the tendency to be undertreated in women due to concerns of drug side effects in terms of both patient and provider levels in clinical practice.

### Study limitations

Our study has some limitations. First, although this subgroup analysis was pre-specified, randomization was not stratified by sex, and the subgroups based on sex were not specifically powered for the occurrence of primary or secondary outcomes. Second, the number of female subgroups was modest, and there were significant differences in baseline characteristics between women and men. Despite the multivariate adjustment for baseline characteristic differences, residual confounding may exist. Thus, our findings should be considered as hypothesis-generating results, and a future larger study is required to confirm our results. Third, the study population mostly comprised Korean ethnicity, it is unclear whether the results can be applied equally to Western population. In fact, the maximum dose of rosuvastatin in clinical practice is 20 mg per day in Korea, while the dose of 40 mg per day in the Western countries. Genetic polymorphisms affecting rosuvastatin pharmacokinetics are more common in Asian than in Western population, and racial differences in response to statins have been reported^29^, requiring additional research for generalization for other ethnic population. Fourth, the RACING trial is an open-label trial. Physicians and patients were not blinded to the group assignments, which may have led to a bias in reporting patient symptoms. Fifth, some components of efficacy outcomes were difficult to compare because of the small number of events. Sixth, although the fourth universal definition of MI no longer includes creatine kinase MB^30^, elevated creatine kinase MB was included as a part of the definition of MI in the RACING trial and troponin-based analysis was not available.

## Conclusions

This prespecified sex-based subgroup analysis showed that the effect of ezetimibe in combination with moderate-intensity statin therapy was not inferior to that of high-intensity statin monotherapy in terms of a 3-year composite of cardiovascular death, major cardiovascular events, or non-fatal stroke in both women and men. Better lipid-lowering effects and safety outcomes of ezetimibe combination therapy compared to high-intensity statin monotherapy were observed irrespective of sex. These findings suggest that the use of ezetimibe in combination with moderate-intensity statin therapy can be an alternative to high-intensity statin monotherapy without sex-specific differentiation of treatment strategies when the use of high-intensity statins is intolerable or the target goal of LDL cholesterol levels cannot be achieved.

### Supplementary Information

Supplementary Information.

## Data Availability

The data that support the findings of this study are available from the corresponding author upon reasonable request.

## Abbreviations

ASCVD: Atherosclerotic cardiovascular disease
IMPROVE-IT: Improved Reduction of Outcomes: Vytorin Efficacy International Trial
LDL: Low-density lipoprotein
MI: Myocardial infarction
RACING: Randomized Comparison of Efficacy and Safety of Lipid-lowering with Statin Monotherapy Versus Statin/ezetimibe Combination for High-risk Cardiovascular Disease
