# Supplementary material for: Effect of moderate-intensity statin with ezetimibe combination vs. high-intensity statin therapy according to sex in patients with atherosclerosis
Source: Sci Rep. 2023 Nov 17;13:20157. doi: 10.1038/s41598-023-47505-x (PMC10656546; doi:10.1038/s41598-023-47505-x)
Supplement: Supplementary file 1 — Supplementary Information. [file 41598_2023_47505_MOESM1_ESM.docx]

**Supplementary materials**

**Byung Gyu Kim, et al.** **Effect of Moderate-Intensity Statin With Ezetimibe Combination vs.High-intensity Statin Therapy According to Sex in Patients With Atherosclerosis**

**Contents**

**I. Supplementary Figures**

Figure S1. The 3-year clinical outcomes by sex

Figure S2. Low-density lipoprotein (LDL) cholesterol concentrations over time by sexes

Figure S3. Time-to-event curves of the atherosclerotic cardiovascular events in women and men

**II.** **Supplementary Tables**

Table S1. Baseline characteristics according to sex and treatment strategies

Table S2. Secondary safety outcomes by sex

Table S3. Adjusted risk of primary and secondary efficacy outcomes by sex and therapy strategy

Table S4. Proportion of patients with LDL cholesterol levels <55 mg/dL by sex and therapy strategy

Table S5. Serial changes in other lipid profiles by sex and therapy strategy

**III. RACING Trial Study Protocol**

**I. Supplementary Figures**

**Figure S1. The 3-year clinical outcomes by sex**

**
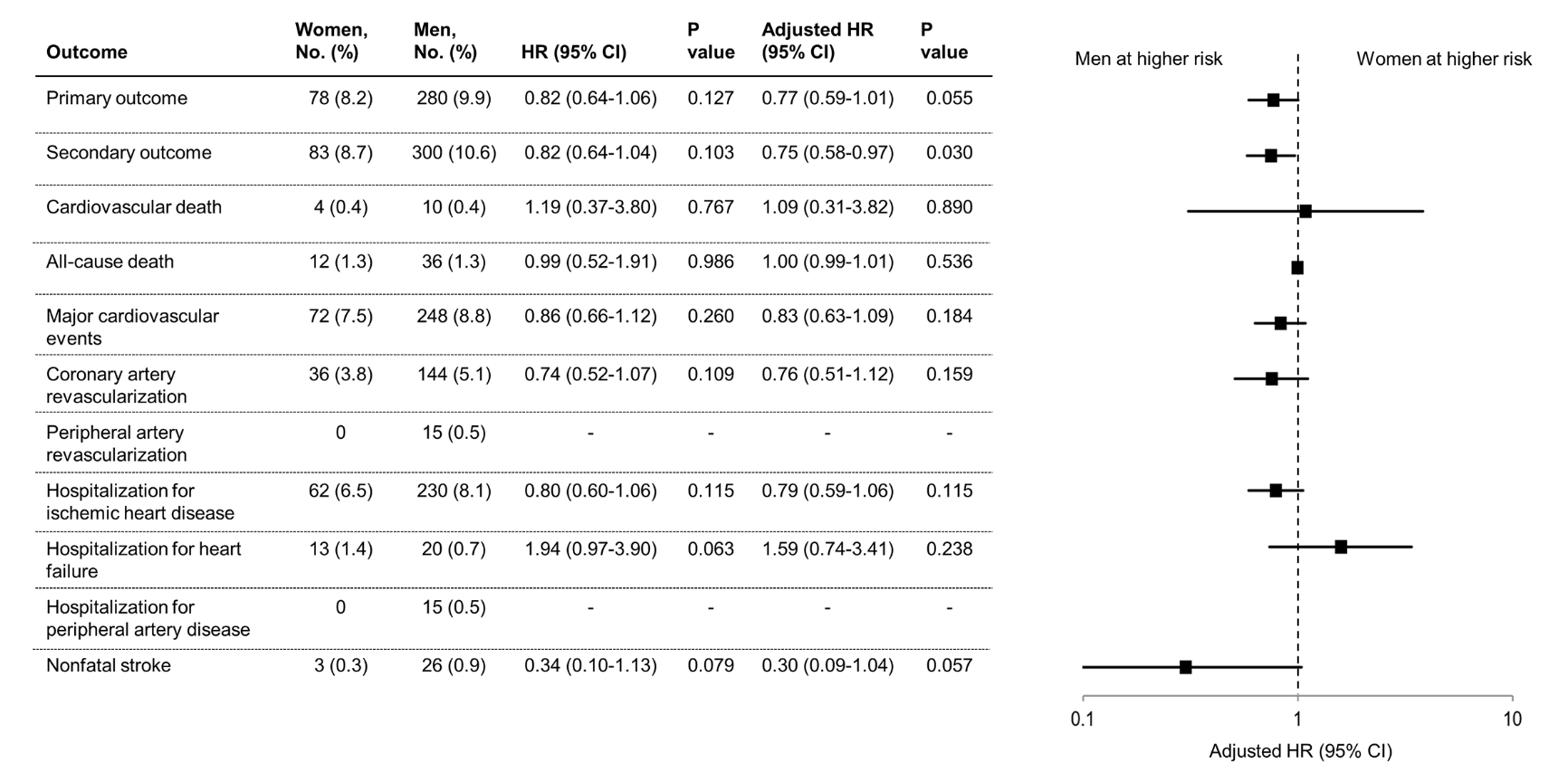
**

Men were used as the reference category. Adjusted hazard ratios (HR) were adjusted for age, body mass index, prior myocardial infarction, prior percutaneous coronary artery intervention, hypertension, chronic kidney disease, current smoker, and baseline low density lipoprotein cholesterol level.

**Figure S2. Low-density lipoprotein (LDL) cholesterol concentrations over time according to sexes**

**
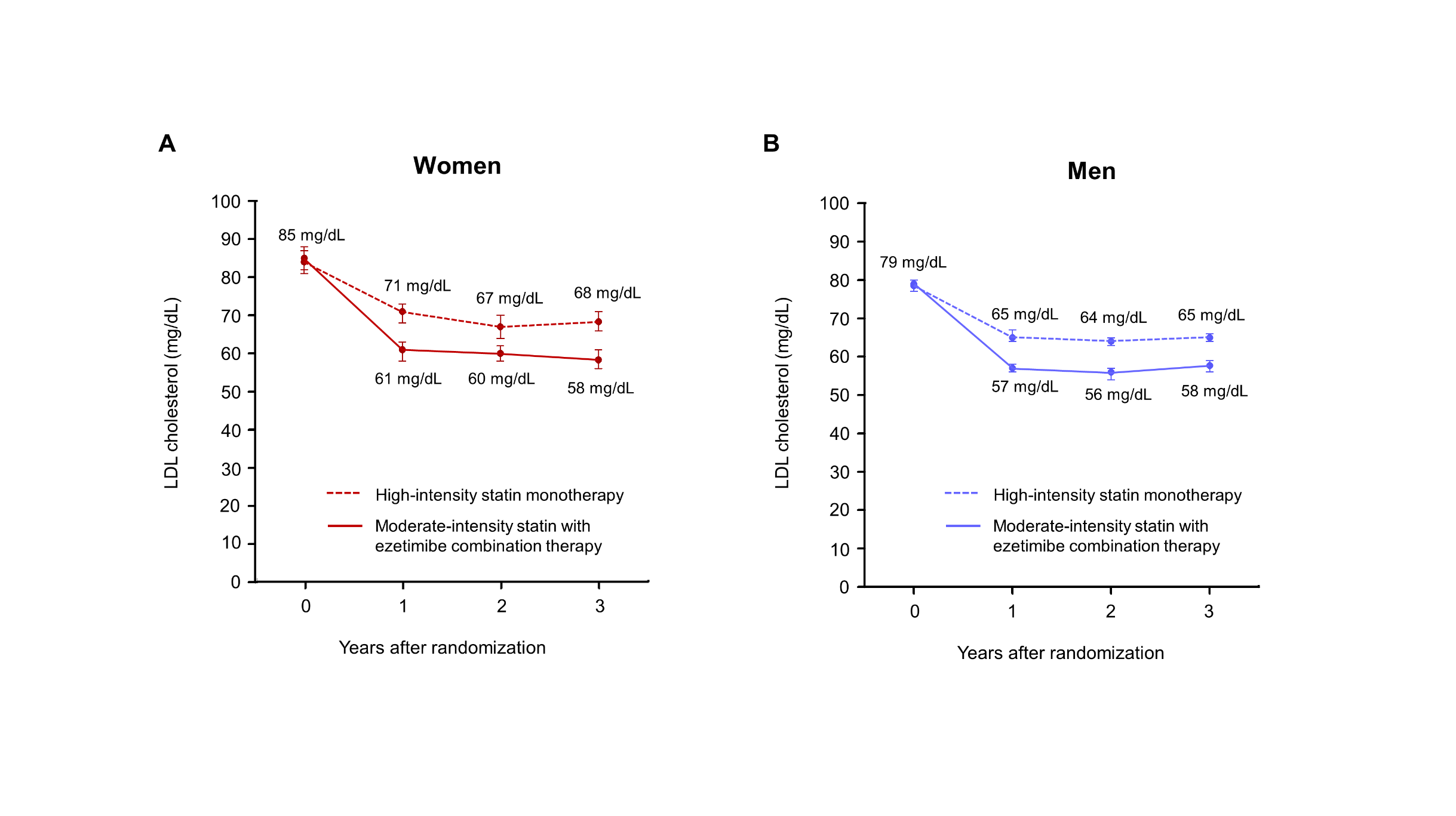
**

Serial median values of LDL cholesterol in women (A) and men (B). The I-bar indicates 95% confidence intervals. Under the graph, values for the comparison between the ezetimibe combination therapy group and the high-intensity statin monotherapy group in LDL cholesterol levels at 1, 2, and 3 years are presented.

**Figure S3. Time-to-event curves of the atherosclerotic cardiovascular events in women and men**

**
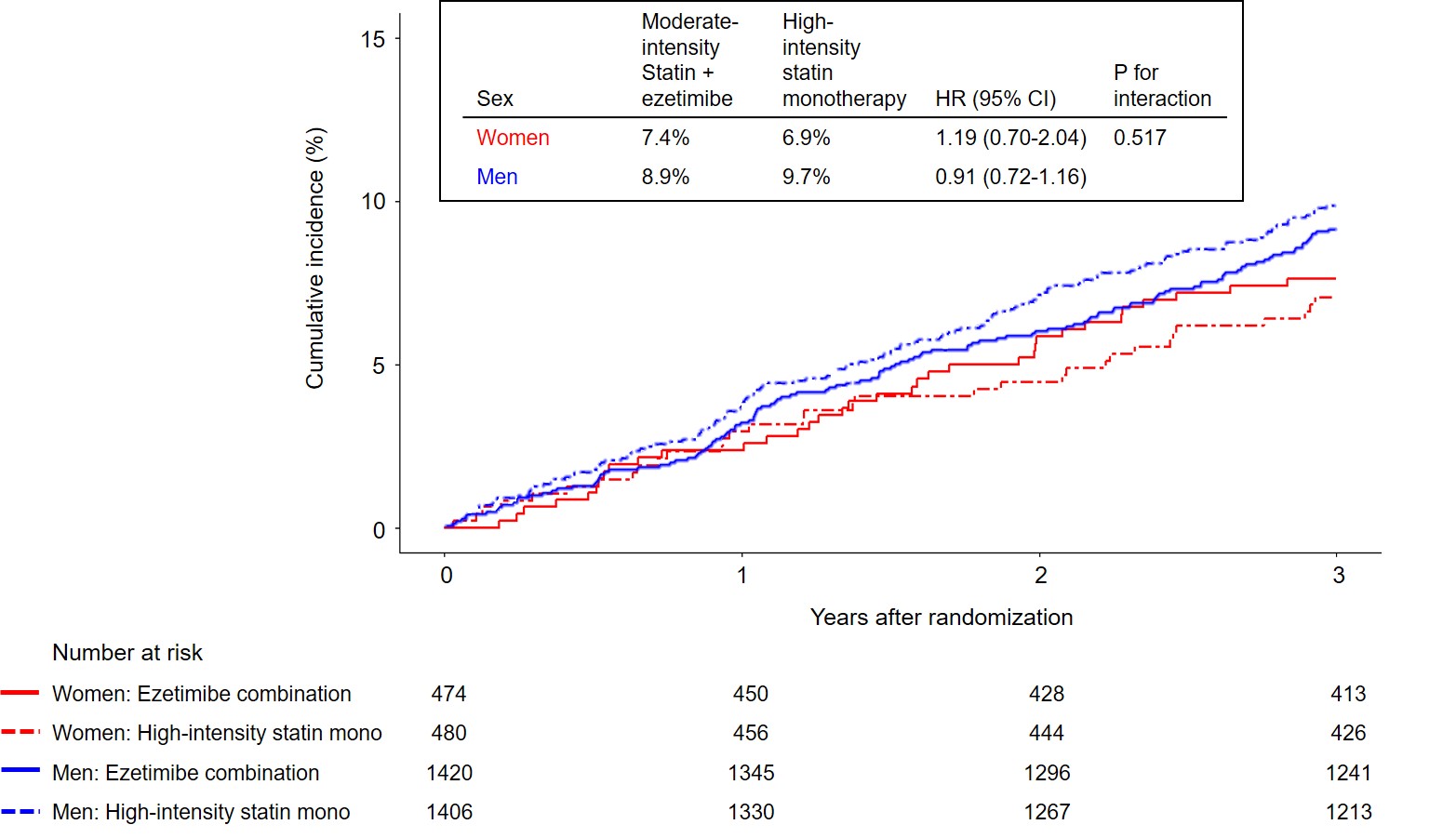
**

Kaplan-Meier curves for atherosclerotic cardiovascular events (a composite of cardiovascular death, coronary or peripheral revascularization, hospitalization for ischemic heart disease or peripheral artery disease, and non-fatal stroke) excluding hospitalization for heart failure according to sexes and treatment assignment. CI, confidence interval; HR, hazard ratio

**II. Supplementary Tables**

**Table S1. Baseline characteristics according to sex and treatment strategies**

| Characteristics | Women (n=954) | | | Men (n=2826) | | |
| --- | --- | --- | --- | --- | --- | --- |
|  | Ezetimibe combination therapy (n=474) | High-intensity statin monotherapy (n=480) | P value | Ezetimibe combination therapy (n=1420) | High-intensity statin monotherapy (n=1406) | P value |
| Age, years | 67.1 ± 8.4 | 67.8 ± 8.5 | 0.219 | 62.4 ± 9.6 | 62.8 ± 9.7 | 0.279 |
| Weight, kg | 59.5 ± 9.0 | 59.9 ± 8.8 | 0.473 | 71.4 ± 10.5 | 71.0 ± 10.2 | 0.350 |
| Body mass index, kg/m^2^ | 24.7 ± 3.5 | 25.0 ± 3.4 | 0.227 | 25.1 ± 3.0 | 25.1 ± 2.9 | 0.918 |
| Previous myocardial infarction | 122 (25.7) | 129 (26.9) | 0.745 | 622 (43.8) | 616 (43.8) | 1.000 |
| Previous percutaneous coronary intervention | 264 (55.7) | 268 (55.8) | 1.000 | 994 (70.0) | 971 (69.1) | 0.616 |
| Previous coronary bypass graft surgery | 26 (5.5) | 28 (5.8) | 0.926 | 106 (7.5) | 87 (6.2) | 0.204 |
| Acute coronary syndrome | 5 (1.1) | 6 (1.2) | 1.000 | 22 (1.5) | 14 (1.0) | 0.253 |
| Previous ischemic stroke | 25 (5.3) | 31 (6.5) | 0.522 | 76 (5.4) | 81 (5.8) | 0.695 |
| Chronic kidney disease^a^ | 55 (11.6) | 72 (15.0) | 0.147 | 138 (9.7) | 127 (9.0) | 0.575 |
| End-stage kidney disease on dialysis | 4 (0.8) | 6 (1.2) | 0.766 | 9 (0.6) | 10 (0.7) | 0.983 |
| Peripheral artery disease | 19 (4.0) | 18 (3.8) | 0.969 | 47 (3.3) | 51 (3.6) | 0.720 |
| Hypertension | 329 (69.4) | 347 (72.3) | 0.364 | 917 (64.6) | 927 (65.9) | 0.474 |
| Diabetes | 156 (32.9) | 182 (37.9) | 0.122 | 545 (38.4) | 515 (36.6) | 0.356 |
| Diabetes with insulin | 14 (3.0) | 20 (4.2) | 0.403 | 36 (2.5) | 50 (3.6) | 0.141 |
| Current smoker | 15 (3.2) | 14 (2.9) | 0.973 | 313 (22.0) | 296 (21.1) | 0.553 |
| Medication for dyslipidemia before randomization |  |  | 0.013 |  |  | 0.107 |
| High-intensity statin | 172 (36.3) | 152 (31.7) |  | 539 (38.0) | 577 (41.0) |  |
| High-intensity statin with ezetimibe | 21 (4.4) | 15 (3.1) |  | 64 (4.5) | 48 (3.4) |  |
| Moderate-intensity statin | 175 (36.9) | 181 (37.7) |  | 506 (35.6) | 504 (35.8) |  |
| Moderate-intensity statin with ezetimibe | 63 (13.3) | 102 (21.2) |  | 188 (13.2) | 146 (10.4) |  |
| Low-intensity statin | 1 (0.2) | 0 |  | 5 (0.4) | 5 (0.4) |  |
| None | 42 (8.9) | 30 (6.2) |  | 118 (8.3) | 126 (9.0) |  |
| Serum LDL cholesterol concentration, mg/dL | 89.4 ± 31.8 | 90.4 ± 33.6 | 0.648 | 83.3 ± 30.5 | 83.7 ± 30.9 | 0.722 |
| Number of patients with LDL cholesterol concentration <70mg/dL | 135 (28.5) | 125 (26.0) | 0.439 | 508 (35.8) | 491 (34.9) | 0.664 |

Data are mean ± SD, median (interquartile range), or number (%). LDL, low-density lipoprotein ^a^Chronic kidney disease was defined as an estimated glomerular filtration rate of less than 60 ml per min per 1.73 m^2^ of body-surface area.

**Table S2. Secondary safety outcomes by sex**

|  | Women (n = 929) | Men (n = 2,749) | P value |
| --- | --- | --- | --- |
| Discontinuation or dose reduction of the study drug due to intolerance | 61 (6.6) | 177 (6.4) | 0.891 |
| Patients’ reported symptoms |  |  |  |
| Dizziness or general weakness | 8 (0.9) | 23 (0.8) |  |
| Chest discomfort or headache | 4 (0.4) | 15 (0.6) |  |
| Gastrointestinal symptom | 2 (0.2) | 11 (0.4) |  |
| Urticaria or itching sensation | 6 (0.6) | 7 (0.3) |  |
| Myalgia | 8 (0.9) | 21 (0.8) |  |
| Other | 6 (0.6) | 2 (0.1) |  |
| Physicians’ discretion |  |  |  |
| Liver enzyme elevation | 10 (1.1) | 37 (1.4) |  |
| Creatine kinase elevation | 12 (1.3) | 46 (1.7) |  |
| Fasting glucose level elevation | 3 (0.3) | 8 (0.3) |  |
| Other | 2 (0.2) | 7 (0.3) |  |
| New-onset diabetes | 110 (11.8) | 289 (10.5) | 0.261 |
| New-onset diabetes with initiation of anti-diabetic medication | 45 (4.8) | 157 (5.7) | 0.316 |
| Muscle-related adverse events | 12 (1.3) | 43 (1.6) | 0.555 |
| Myalgia | 10 (1.1) | 36 (1.3) | 0.581 |
| Myopathy | 0 | 6 (0.2) | 0.992 |
| Myonecrosis^a^ | 3 (0.3) | 21 (0.8) | 0.162 |
| Mild | 1 (0.1) | 16 (0.6) |  |
| Moderate | 1 (0.1) | 4 (0.2) |  |
| Severe including rhabdomyolysis | 1 (0.1) | 1 |  |
| Gallbladder-related adverse events | 2 (0.2) | 17 (0.6) | 0.157 |
| Major bleeding | 5 (0.5) | 23 (0.8) | 0.369 |
| Cancer diagnosis | 10 (1.1) | 53 (1.9) | 0.088 |
| New-onset neurocognitive disorder | 2 (0.2) | 4 (0.1) | 0.651 |
| Cataract surgery | 10 (1.1) | 30 (1.1) | 0.970 |

Data are number (%).

^a^Severity of myonecrosis was classified by an elevation of creatine kinase level compared with either baseline level or the upper limit of normal (ULN); mild, 3–10 times ULN; moderate, 10–50 times ULN; severe, >50 times ULN.

**Table S3. Adjusted risk of primary and secondary efficacy outcomes by sex and therapy strategy**

|  | Women  (n=954) | | | |  | Men  (n=2826) | | | |  |
| --- | --- | --- | --- | --- | --- | --- | --- | --- | --- | --- |
|  | Ezetimibe combination therapy (n=474) | High-intensity statin monotherapy (n=480) | Adjusted HR (95% CI)^a^ | P-value |  | Ezetimibe combination therapy (n=1420) | High-intensity statin monotherapy (n=1406) | Adjusted HR (95% CI)^a^ | P-value |  |
| **Primary outcome** | | | | | | | | | | |
| Composite of cardiovascular death, major cardiovascular events, or nonfatal stroke | 38 (8.0) | 40 (8.3) | 1.02 (0.65-1.59) | 0.942 |  | 134 (9.4) | 146 (10.4) | 0.91 (0.72-1.16) | 0.462 |  |
| **Secondary outcome** | | | | | | | | | | |
| Composite of all-cause death, major cardiovascular events, or nonfatal stroke | 41 (8.6) | 42 (8.8) | 1.06 (0.69-1.64) | 0.784 |  | 145 (10.2) | 155 (11.0) | 0.93 (0.74-1.17) | 0.558 |  |
| **Individual clinical outcome** | | | | | | | | | | |
| Cardiovascular death | 2 (0.4) | 2 (0.4) | - | - |  | 6 (0.4) | 4 (0.3) | 1.51 (0.43-5.35) | 0.525 |  |
| All-cause death | 6 (1.3) | 6 (1.2) | - | - |  | 20 (1.4) | 16 (1.1) | 1.27 (0.66-2.46) | 0.472 |  |
| Major cardiovascular events | 35 (7.4) | 37 (7.7) | 0.99 (0.62-1.58) | 0.970 |  | 118 (8.3) | 130 (9.2) | 0.90 (0.70-1.16) | 0.432 |  |
| Coronary artery revascularization | 18 (3.8) | 18 (3.8) | 1.08 (0.56-2.09) | 0.815 |  | 73 (5.1) | 71 (5.0) | 1.03 (0.74-1.42) | 0.878 |  |
| Percutaneous coronary intervention | 17 (3.6) | 18 (3.8) |  |  |  | 70 (4.9) | 71 (5.0) |  |  |  |
| Coronary artery bypass surgery | 1 (0.2) | 0 |  |  |  | 3 (0.2) | 0 |  |  |  |
| Peripheral artery revascularization | 0 | 0 | - | - |  | 8 (0.6) | 7 (0.5) | 1.14 (0.41-3.16) | 0.799 |  |
| Hospitalization for ischemic heart disease | 32 (6.8) | 30 (6.2) | 1.12 (0.68-1.85) | 0.660 |  | 110 (7.7) | 120 (8.5) | 0.91 (0.70-1.18) | 0.485 |  |
| Stable angina or unstable angina | 27 (5.7) | 27 (5.6) |  |  |  | 93 (6.5) | 106 (7.5) |  |  |  |
| Acute myocardial infarction | 5 (1.1) | 3 (0.6) |  |  |  | 17 (1.2) | 14 (1.0) |  |  |  |
| Hospitalization for heart failure | 6 (1.3) | 7 (1.5) | 0.97 (0.32-2.91) | 0.955 |  | 8 (0.6) | 12 (0.9) | 0.67 (0.27-1.66) | 0.390 |  |
| Hospitalization for peripheral artery disease | 0 | 0 | - | - |  | 8 (0.6) | 7 (0.5) | 1.14 (0.41-3.15) | 0.800 |  |
| Nonfatal stroke | 2 (0.4) | 1 (0.2) | - | - |  | 13 (0.9) | 13 (0.9) | 0.97 (0.45-2.11) | 0.948 |  |
| Ischemic stroke | 1 (0.2) | 1 (0.2) |  |  |  | 10 (0.7) | 10 (0.7) |  |  |  |
| Hemorrhagic stroke | 1 (0.2) | 0 |  |  |  | 3 (0.2) | 3 (0.2) |  |  |  |

Data are number (% of the cumulative rates at 3 years according to Kaplan-Meier event rates). CI, confience interval; HR, hazard ratio.

^a^Model adjusted for age, body mass index, prior myocardial infarction, prior percutaneous coronary artery intervention, hypertension, chronic kidney disease, current smoker, and baseline low density lipoprotein cholesterol level.

**Table S4. Proportion of patients with LDL cholesterol levels <55 mg/dL by sex and therapy strategy**

|  | Women (n=954) | | |  | Men (n=2826) | | | P-value for interaction^a^ |
| --- | --- | --- | --- | --- | --- | --- | --- | --- |
|  | Ezetimibe combination therapy | High-intensity statin monotherapy | P-value |  | Ezetimibe combination therapy | High-intensity statin monotherapy | P-value |  |
| **Baseline** |  |  |  |  |  |  |  |  |
| Number of patients | 474 | 480 |  |  | 1420 | 1406 |  |  |
| Number of patients with LDL cholesterol levels <55 mg/dL (%) | 48 (10.1) | 48 (10.0) | 1.000 |  | 207 (14.6) | 198 (14.1) | 0.748 | - |
| **1 year** |  |  |  |  |  |  |  |  |
| Number of patients | 428 | 434 |  |  | 1247 | 1239 |  |  |
| Number of patients with LDL cholesterol levels <55 mg/dL (%) | 157 (36.7) | 89 (20.5) | P<0.001 |  | 538 (43.1) | 326 (26.3) | P<0.001 | 0.756 |
| **2 years** |  |  |  |  |  |  |  |  |
| Number of patients | 391 | 389 |  |  | 1167 | 1148 |  |  |
| Number of patients with LDL cholesterol levels <55 mg/dL (%) | 157 (40.2) | 92 (23.7) | P<0.001 |  | 551 (47.2) | 359 (31.2) | P<0.001 | 0.600 |
| **3 years** |  |  |  |  |  |  |  |  |
| Number of patients | 339 | 335 |  |  | 1010 | 979 |  |  |
| Number of patients with LDL cholesterol levels <55 mg/dL (%) | 136 (40.1) | 63 (18.8) | P<0.001 |  | 427 (42.3) | 267 (27.3) | P<0.001 | 0.051 |

Data are median (interquartile range) or number (%).LDL, low-density lipoprotein.

^a^P-value for interaction between sex and therapy.

**Table S5. Serial changes in other lipid profiles by sex and therapy strategy**

|  | Women (n=954) | | |  | Men (n=2826) | | |
| --- | --- | --- | --- | --- | --- | --- | --- |
|  | Ezetimibe combination therapy | High-intensity statin monotherapy | P-value |  | Ezetimibe combination therapy | High-intensity statin monotherapy | P-value |
| **Baseline** |  |  |  |  |  |  |  |
| Number of patients | 474 | 480 |  |  | 1420 | 1406 |  |
| Total cholesterol level, mg/dL | 156 (136–180) | 157 (137–179) | 0.366 |  | 145 (126–168) | 145 (125–170) | 0.545 |
| Triglyceride level, mg/dL | 123 (89–169) | 121 (88–159) | 0.551 |  | 120 (89–167) | 124 (90–167) | 0.911 |
| HDL cholesterol level, mg/dL | 51 (42–58) | 50 (44–59) | 0.166 |  | 45 (39–52) | 45 (38–52) | 0.953 |
| **1 year** |  |  |  |  |  |  |  |
| Number of patients | 428 | 434 |  |  | 1247 | 1239 |  |
| Total cholesterol level, mg/dL | 129 (115–145) | 142 (126–160) | <0.001 |  | 121 (107–138) | 132 (116–148) | <0.001 |
| Triglyceride level, mg/dL | 104 (80–143) | 119 (87–156) | 0.001 |  | 109 (81–151) | 121 (90–165) | 0.002 |
| HDL cholesterol level, mg/dL | 51 (44–58) | 51 (44–59) | 0.841 |  | 45 (39–51) | 45 (39–52) | 0.217 |
| **2 years** |  |  |  |  |  |  |  |
| Number of patients | 391 | 389 |  |  | 1167 | 1148 |  |
| Total cholesterol level, mg/dL | 132 (116–149) | 142 (127–162) | <0.001 |  | 122 (108–139) | 132 (117–150) | <0.001 |
| Triglyceride level, mg/dL | 113 (86–149) | 118 (86–164) | 0.156 |  | 109 (82–151) | 120 (88–165) | 0.004 |
| HDL cholesterol level, mg/dL | 52 (44–60) | 51 (44–60) | 0.586 |  | 45 (39–53) | 46 (39–54) | 0.356 |
| **3 years** |  |  |  |  |  |  |  |
| Number of patients | 339 | 335 |  |  | 1010 | 979 |  |
| Total cholesterol level, mg/dL | 130 (114–145) | 143 (126–159) | <0.001 |  | 123 (109–139) | 133 (118–150) | <0.001 |
| Triglyceride level, mg/dL | 106 (82–138) | 119 (91–159) | 0.011 |  | 111 (81–149) | 118 (88–164) | 0.028 |
| HDL cholesterol level, mg/dL | 50 (42–58) | 51 (43–59) | 0.313 |  | 44 (38–51) | 45 (39–53) | 0.019 |

Data are median (interquartile range). HDL, high-density lipoprotein.

III. Study Protocol

RAndomized Comparison of Efficacy and Safety of Lipid-lowerING With Statin Monotherapy Versus Statin/Ezetimibe Combination for High-risk Cardiovascular Diseases: RACING Trial

Principle Investigator:

Myeong-Ki Hong, MD, PhD

**Myeong-Ki Hong, MD, PhD**

Division of Cardiology, Severance Hospital,

Yonsei University College of Medicine,

Seodaemun-gu, Shinchondong, Seoul, South Korea 120-752

Telephone: +82-2-2228-8445

Fax: +82-2-393-2041

E-mail: mkhong61@ yuhs.ac

**CONTENTS**

**I. Study Protocol**∙∙∙∙∙∙∙∙∙∙∙∙∙∙∙∙∙∙∙∙∙∙∙∙∙∙∙∙∙∙∙∙∙∙∙∙∙∙∙∙∙∙∙∙∙∙∙∙∙∙∙∙∙∙∙∙∙∙∙∙∙∙∙∙∙∙∙∙∙∙∙∙∙∙∙∙∙∙∙∙∙∙∙∙∙∙∙∙∙∙∙∙∙∙∙∙∙∙∙∙∙∙∙∙∙∙∙∙∙∙∙∙∙∙∙∙∙ 4

**II. Summary of Changes** ∙∙∙∙∙∙∙∙∙∙∙∙∙∙∙∙∙∙∙∙∙∙∙∙∙∙∙∙∙∙∙∙∙∙∙∙∙∙∙∙∙∙∙∙∙∙∙∙∙∙∙∙∙∙∙∙∙∙∙∙∙∙∙∙∙∙∙∙∙∙∙∙∙∙∙∙∙∙∙∙∙∙∙∙∙∙∙∙∙∙∙∙ 26

**PROTOCOL FOR THE RACING**

**1. Protocol Summary**

**2. Background**

**3. Study Objectives and Endpoint**

3.1. Primary Endpoint

3.2. Secondary Endpoint

**4. Methods and Design**

4.1. Study Patients

4.2. Sample Size Calculation

4.3. Statistical Analyses Plan

**5. Study Procedure**

5.1. Subject screening, consent, and randomization

5.2. Study Drug

5.3. Follow-up

5.4. General guideline for concomitant treatment

5.5. Protocol of the study at a glance

**6. Study Quality Management**

6.1. Ethical issue

6.2. Data and safety monitoring

6.3. Informed consent

6.4. Safety management

**7. Study Definition**

**8. References**

**1. Protocol Summary**

| Trial Name | RAndomized Comparison of Efficacy and Safety of Lipid-lowerING With Statin Monotherapy Versus Statin/Ezetimibe Combination for High-risk Cardiovascular Diseases: RACING Trial |
| --- | --- |
| Main Center | Severance Hospital, Yonsei University College of Medicine |
| Trial Phase | Phase IV |
| Objective | To compare the clinical efficacy and safety of combination therapy with moderate-intensity statin and ezetimibe versus high-intensity statin monotherapy in patients with high-risk cardiovascular diseases |
| Method | Prospective, multicenter, randomized, open-label study |
| Number of patients | 3780 high-risk cardiovascular disease requiring high intensity statin |
| Study Design | - Prospective, open label, randomized, multicenter study - Patients will be randomized 1:1 to either of ezetimibe/moderate-intensity statin combination therapy or high-intensity statin monotherapy - A stratification of LDL-cholesterol and diabetes will be used for an randomization. - Patients will be followed-up for 36 months. |
| Main Inclusion Criteria | - Age 19−80 years - High-risk cardiovascular disease (meeting at least one): Previous myocardial infarction, acute coronary syndrome (Unstable angina or myocardial infarction), coronary revascularization or other arterial revascularization procedures, ischemic stroke, peripheral artery disease |
| Study Endpoint | - Primary Endpoint: Composite of cardiovascular death, major cardiovascular event (coronary or peripheral revascularization, or hospitalization for cardiovascular events), or nonfatal stroke within 3 years - Secondary Endpoint:   1) Proportion of patients with LDL-cholesterol <70 mg/dL at 1, 2, and 3 years  2) Composite of all death, major cardiovascular event, or nonfatal stroke  3) Discontinuation or dose-reduction of study drug by intolerance  4) Clinical adverse events (new-onset diabetes mellitus, muscle-related adverse events, gastrointestinal symptoms, gallbladder-related adverse events, major bleeding, cancer diagnosis, new-onset neurocognitive disorder, or cataract surgery) |
| Statistical Methods | Cumulative incidence using Kaplan-Meier method  Log-rank test  Cox proportional hazard regression model |
| Study Duration | Overall study will be completed in Jan 4^th^, 2023, including 2 years of recruitment and the duration of clinical follow-up and data analyses. |
| Participating Sites | 26 centers including Severance Hospital  50-1 Yonsei-ro, Seodaemun-gu, Shinchondong, Seoul, South Korea 03722 |

**2. BACKGROUND**

The major treatable causes of atherosclerotic cardiovascular disease (ASCVD) include hypercholesterolemia, hypertension, diabetes, and an unhealthy lifestyle. Because low-density lipoprotein-cholesterol (LDL-C) plays a significant role in the promotion, development, and progression of vascular atherosclerosis, a primary strategy in these efforts has been lowering of LDL-C in at-risk populations. Statin, has beneficial properties include atherosclerotic plaque stabilization, oxidative stress reduction, enhancement of endothelial function and a decrease in vascular inflammation beyond their lipid-lowering effect. In various clinical trials, statins have shown clinical benefits in primary and secondary prevention. Epidemiological studies and recent randomized clinical trials demonstrate a continuous relationship between cholesterol levels and ASCVD risk: the more LDL-C is lowered, the greater the risk reduction. In addition, most recent study, IMPROVE-IT proved clinical efficacy of additive ezetimibe which targets the Niemann–Pick C1–like 1 (NPC1L1) protein, thereby reducing absorption of cholesterol from the intestine. Ezetimibe with simvastatin was effective to reduce clinical events with lower LDL-C in patients with acute coronary syndrome. Thus, it can be said that “the lower, the better” is true for cholesterol reduction.

The NCEP ATP III guideline and 2004 update have served as the standard of care for at-risk patients with hyperlipidemia for nearly a decade. Guideline focused on the fasting lipid panel as the initial evaluation of lipid-related CVD risk. Within each category of ASCVD risk, targets of treatment are then specified in these recommendations. In the ATP III guidelines, cardiovascular disease (CVD) and diabetes mellitus as a coronary heart disease risk equivalent were considered as high-risk category. LDL-C was considered the primary target of therapy and an optional goal of LDL-C <70 mg/dl in these high–risk patients. The European Society of Cardiology and the European Atherosclerosis Society guidelines for the management of dyslipidemias define documented cardiovascular disease, previous myocardial infarction, coronary revascularization, ischemic stroke, DM with target organ damage, or moderate to severe CKD as very high risk group and recommended target LDL -C level of <70mg/dL and/or ≥50% LDL-C reduction. Contrast to previous guidelines focused on targeting LDL-C level for optimal treatment in high-risk patients, most recent ACC/AHA Guideline on the Treatment of Blood Cholesterol to Reduce Atherosclerotic Cardiovascular Risk in Adult in 2013 suggested that primary focus of treatment in such high-risk patients should be *intensity-directed statin treatment itself*. On the basis of evidence, 4 major statin benefit groups were identified: 1) with clinical ASCVD, 2) primary elevations of LDL-C ≥190 mg/dL, 3) diabetes age 40-75 years with LDL-C 70-189 and without clinical ASCVD, or 4) estimated 10-year ASCVD risk ≥7.5%. For these groups, the new guideline proposes that implementation of cholesterol-lowering treatment using evidenced-based intensity of statin therapy should be treated in patients without such targets.

The clinical efficacy of LDL-lowering therapy have been proven with strong evidences and more emphasized. However, there are also growing concerns that high-intensity statin would be related to increased risk of adverse effects. For example, statin therapy modestly increases the risk for developing type 2 diabetes. Conventional strategies for lowering LDL-cholesterol was focused on statins, therefore doubling of previously described dose of statin would be common way in patients with inadequate lowering LDL-cholesterol level. Additive ezetimibe will also an alternative strategy not only to lower LDL-cholesterol level and also to reduce the need of dosage of high-intensity statin to fulfill sufficient LDL-cholesterol lowering effect. However, these two different treatment strategies will be more evaluated in regard to lipid-lowering efficacy and clinical outcomes including occurrence of cardiovascular events.

We will evaluate whether additive ezetimibe with rosuvastatin will have comparable clinical efficacy in terms of clinical outcomes and goal attainment of LDL-C compared to rosuvastatin monotherapy in patients with high-risk cardiovascular disease.

**3. Study Objectives and Endpoint**

To compare the clinical efficacy and safety of combination therapy with moderate-intensity statin and ezetimibe versus high-intensity statin monotherapy in patients with high-risk cardiovascular diseases

**3.1. Primary Endpoint**

The occurrence of cardiovascular death, major cardiovascular events, or nonfatal stroke within 3 years. Major cardiovascular events included coronary or peripheral revascularization and hospitalization for cardiovascular events

**3.2. Secondary Endpoint**

1. Proportion of patients with LDL-cholesterol <70 mg/dL at 1, 2, and 3 years

2. Composite of all death, major cardiovascular event, or nonfatal stroke

3. Discontinuation or dose-reduction of study drug by intolerance

4. Clinical adverse events (new-onset diabetes mellitus, muscle-related adverse events, gastrointestinal symptoms [dyspepsia or abdominal pain not explainable with other cause], gallbladder-related adverse events, major bleeding, cancer diagnosis, new-onset neurocognitive disorder, or cataract surgery)

**4. Methods and Design**

**4.1. Study Patients**

**4.1.1. Inclusion Criteria**

1. Age 19 − 80 years

2. High-risk cardiovascular disease (meeting at least one):

- 1. Previous myocardial infarction
  2. Acute coronary syndrome (Unstable angina or myocardial infarction)
  3. Coronary revascularization or other arterial revascularization procedures
  4. Ischemic stroke
  5. Peripheral artery disease

**4.1.2. Exclusion criteria**

1. Active liver disease or persistent unexplained serum AST or ALT elevation more than 2 times the upper limit of normal range

2. Allergy or hypersensitivity to any statin or ezetimibe

3. Solid organ transplantation recipient

4. History of any adverse drug reaction requiring discontinuation of statin

5. Pregnant women, women with potential childbearing, or lactating women

6. Life expectancy less than 3 years

7. Inability to follow the patient over the period of 1 year after enrollment, as assessed by the investigator

8. Inability to understand or read the informed content

**4.2. Sample Size Calculation**

It will be tested whether the combination therapy group is noninferior to the statin monotherapy group in terms of the primary endpoint at 3 years. We assumed that the expected primary endpoint will occur 13% in the combination therapy group and 14% in the statin monotherapy group, according to the result of IMPROVE-IT trial (Primary endpoint: 34.7% vs. 32.7% in simvastatin vs. simvastatin plus ezetimibe group with 6 years of mean follow-up duration). A non-inferiority margin of 2.0% is selected. With a one-sided type I error of 0.05 and 80% power, a sample size of 1605 patients in each arm is required (total 3210 patients). Assuming around 15% loss to follow-up, a total of 3780 patients will be randomized. The sample size determination was based on a pure frequency analysis, whereas the endpoint analysis will use the Kaplan-Meier estimates. Since the two methods are equivalent in the absence of censoring, and a sufficient number of uncensored patients is anticipated, the sample size should be adequate.

Attainment with LDL-cholesterol less than 70 mg/dL at 1 year was 30.5% vs. 50.6% in simvastatin vs. simvastatin plus ezetimibe group from IMPROVE-IT. LDL-cholesterol lowering effect of simvastatin 40 mg plus ezetimibe 10 mg was known to be similar to rosuvastatin 20 mg. Therefore, we assumed that goal attainment with lowering LDL-cholesterol would be about 50% vs. 70%. With superiority hypothesis with 5% alpha error rate, 80% power and estimated 15% of loss to follow-up, a total of 220 patients were required, which was sufficiently fulfilled by the sample size of 3780 patients as per the primary objective of this trial, suggesting sufficient power.

**4.3. Statistical Analyses Plan**

**4.3.1. Analysis of the Primary Endpoint**

Our hypothesis is that the combination therapy will be non-inferior to the statin monotherapy with the non-inferiority margin of 2.0%. The null hypothesis for this analysis is that the three-year rate of primary endpoint is at least 2.0% higher in the combination therapy group versus statin monotherapy group. The alternative for this analysis is that absolute risk difference at three years is less than 2.0%.

H*_0_*: (Event rate)_combination_ – (Event rate)_monotherapy_ ≥2.0%

H*_A_*: (Event rate)_combination_ – (Event rate)_monotherapy_ <2.0%

The test is performed at a point in time T, using the Kaplan-Meier estimates for freedom from the primary endpoint being evaluated, and the Greenwood standard errors for these estimates. The null hypothesis will be rejected if the upper limit of the 95% CI for the absolute risk difference in the event rate of the primary endpoint at 3 years is less than 2.0%. The primary analysis will be performed according to the intention to treat principle. Analysis of the primary endpoint also will be performed on the per-protocol population. In the per-protocol population, the following patients with protocol deviations will be excluded: (1) The patients found to be ineligible; (2) Informed consent not obtained; (3) Randomized therapy not implemented (a total period of the discontinued the allocated treatment >5% of a total follow-up period).

**4.3.2. Analysis of the Secondary Endpoint**

As for the clinical outcomes during 3 years, cumulative event rate during the clinical follow-up will be estimated using the Kaplan-Meier method and will be compared with the log-rank test. If needed, Cox proportional hazard regression analysis will be used after testing proportional hazard assumption. As for the comparison of the proportions, Chi-square test will be performed.

**4.3.3. Other analyses**

A subgroup analysis will be performed for clinically relevant factors such as age, sex, body mass index, hypertension, diabetes mellitus, chronic kidney disease, previous MI, ACS, stroke, PAD, and baseline LDL-Cholesterol <100 mg/dL.

**5. Study Procedure**

**5.1. Subject screening, consent, and randomization**

All eligible patients who are at high-risk of cardiovascular disease will be screened according to inclusion and exclusion criteria. A qualified member of the investigational site’s research team will review the subject’s medical history and screen for the study eligibility.

All subjects must complete a Subject Informed Consent Form prior to undergoing randomization. In advance of the consent discussion, the subject should receive the IRB-approved Subject Informed Consent Form. During the consent discussion, the investigator or his/her designee must fully inform the subject of all pertinent aspects and risks of the study. All items discussed in the Subject Informed Consent Form must be explained by research site staff. Neither the investigator nor the investigation site staff shall coerce or unduly influence a subject to participate or to continue to participate in the clinical study. The informed consent process shall not waive or appear to waive the subject’s rights. The subject will be provided ample time to read and understand the Subject Informed Consent Form and to consider participation in the study. When the subject decides to participate in the clinical study, the site’s current IRB-approved Subject Informed Consent Form must be signed and personally dated by the subject and investigator designee.

After voluntary agreement with informed consent. The patients will be randomized 1:1 to either of ezetimibe/moderate-intensity statin combination therapy group or high-intensity statin monotherapy. A stratification of the baseline LDL-cholesterol level and diabetes will be performed. Permuted block randomization 4 to 6 will be used for allocation.

**5.2. Study Drug**

| **Categories** | **Study drugs** | **Dosage** |
| --- | --- | --- |
| HMG-CoA reductase inhibitor | Rosuvastatin | 20 mg |
| HMG-CoA reductase inhibitor plus NPC1L1 antagonist | Rosuvastatin plus ezetimibe | Rosuvastatin 10 mg plus ezetimibe 10 mg |

During the study period, the patients who allocated to the combination therapy group will be given rosuvastatin 10 mg with ezetimibe 10 mg, and those who allocated to the statin monotherapy group will be given rosuvastatin 20 mg for 1 year.

After 1 year of randomization, the dose of rosuvastatin can be decreased at physicians’ discretion when the LDL cholesterol level is maintained less than 50 mg/dL and the patients do not have any adverse clinical events. The reasons of the dose reduction of statin should be recorded in detail.

**5.3. Follow-up**

Baseline characteristics, laboratory findings including lipid profiles will be obtained at enrollment. If statin dosage was adjusted, the patient will visit to outpatient clinic and laboratory assessment (lipid profile and liver enzyme) after 4-12 weeks. Clinical check-up with laboratory exam including lipid profile and liver enzyme will be followed at 2, 6 and 12 months after enrollment. After 12 months from enrollment, we will follow clinical check-up and laboratory evaluation will be conducted every year until the end of 3-year follow-up.

**Table. Schedules for follow-up**

| Measurement | Baseline | Follow-up | | | | |
| --- | --- | --- | --- | --- | --- | --- |
|  |  | 8W±4W | 6M±1M | 12M±2M | 24M±2M | 36M±2M |
| Informed consent | O |  |  |  |  |  |
| Inclusion/Exclusion | O |  |  |  |  |  |
| Clinical history | O |  |  |  |  |  |
| Vital sign/ Physical exam | O | O  (option) | O  (option) | O | O | O |
| Height and Weight | O | O  (option) | O  (option) | O | O | O |
| Waist circumference | O |  |  | O | O | O |
| ECG (12 lead) | O |  |  | O | O | O |
| CBC, Routine chemistry, Lipid profile | O^1^ | O^2^  (option) | O^2^  (option) | O | O | O |
| Creatine kinase (CK) ^5^ | O^1^  (option) |  |  | O  (option) |  | O  (option) |
| HbA1C, AC insulin^3^  hs-CRP | O^1^ |  |  | O |  | O |
| Urine protein/creatinine or albumin/creatinine | O  (option) |  |  | O  (option) | O  (option) | O  (option) |
| Pregnancy test (if needed) | O |  |  |  |  |  |
| Medication | O | O | O | O | O | O |
| Clinical adverse events | O | O | O | O | O | O |

1. If the patients who had not given the statin, it is recommended to examine the baseline lab at least 8 weeks before.

2. It is strongly recommended to examine the test 8 weeks and 6 months after the enrollment and after the drug dose changes.

3. If the patients had no diabetes or impaired glucose tolerance, this test will be covered by study fund.

4. If the patients had diabetes or chronic kidney disease, this test will be performed by the current clinical guidelines.

5. If the patients had muscle-relate adverse events, this test is strongly recommended. Otherwise, it will be performed by physicians’ discretion.

**5.4.** **General guideline for concomitant treatment**

Risk factor modification for cardiovascular disease should be initiated for all patients as recommended. All medication except statin will be used according to current guidelines. Non-statin lipid-lowering drugs can be concurrently administered with study drugs at the physician’s discretion.

**5.5. Protocol of the study at a glance**

**
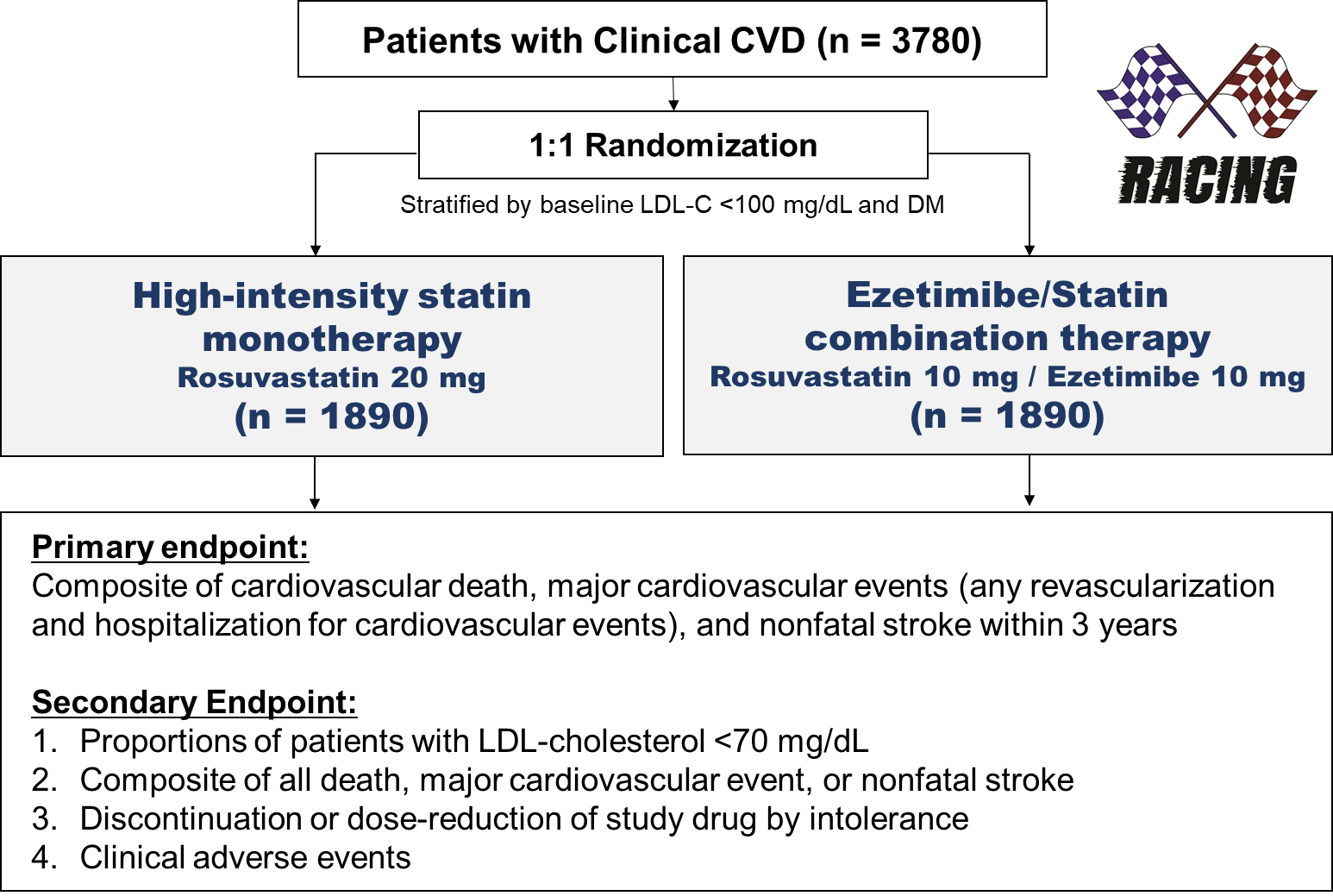
**

**6. Study Quality Management**

**6.1. Ethical Issue**

The primary investigator (PI) has the responsibility to abide by ethical requirements related to this study. This study will be conducted with approval of institutional review board (IRB) and after voluntary agreements with informed consent from all patients. In addition, we disclose that this study is not contrary to Helsinki declaration and ICH/GCP.

Data collected on each subject will be recorded on a web-based eCRF. Each enrolled subject is assigned a unique study ID number. Records of the subject/subject ID relationship will be maintained by the study site. Individual subject medical information obtained as a result of this study will be considered confidential. Authorized site personnel will record the required data on eCRFs. Study personnel delegated for eCRF completion and/or approval will be trained on the use of the eCRF system and thereafter be provided with a user name and password to access the system. Passwords are individual and cannot be shared. The eCRFs must be completed and updated to reflect the latest observations on the subjects participating in the study. The investigator (or approved sub-investigator) will electronically sign the appropriate pages of each eCRF. All study-related data will be stored for 10 years after the complete of the study.

**6.2. Data and safety monitoring**

The Principal Investigator (PI) will be responsible for ensuring participants’ safety. The Data and Safety Monitoring Board (DSMB) will act in an advisory capacity to monitor participant safety, evaluate the progress of the study, to review procedures for maintaining the confidentiality of data, the quality of data collection, management, and analyses.

|  | Centers | Members |
| --- | --- | --- |
| Data Safety Monitoring Board | Yonsei Cardiovascular Hospital | Jae Sun Uhm |
|  | Catholic University, Seoul | Jong-Chan Youn |
|  | Ewha Womans University | Junbeom Park |
|  | Severance Hospital | Dong-Ho Shin |

The PI will be informed of serious adverse events as soon as they occur and will notify the DSMB within 24 hours of notification. DSMB will meet twice annually, either in-person or by teleconference call to review study progress, data quality, and participant’s safety. The content of the data and safety monitoring report will include study status, participant descriptive information, safety information, and study quality

**6.3. Informed consent**

The Principal Investigator will ensure that the patient is given full and adequate oral and written information about the nature, purpose, possible risk and benefit of the study. Patients must also be notified that they are free to discontinue from the study at any time. The patient should be given the opportunity to ask questions and allowed time to consider the information provided. The Principal Investigator must store the original, signed Informed Consent Form. Finally, the investigators will repeatedly confirm the patient’s intention whether to continue or withdraw this study every follow-up.

**6.4. Safety management**

**6.4.1. Definition**

Adverse Event is defined as follows. Any untoward or unfavorable medical occurrence in a human study participant, including any abnormal sign (e.g. abnormal physical exam or laboratory finding), symptom, or disease, temporally associated with the participants’ involvement in the research, whether or not considered related to participation in the research.

Serious Adverse Event is defined as follows. Results in death; life threatening, or places the participant at immediate risk of death from the event as it occurred; requires or prolongs hospitalization; causes persistent or significant disability or incapacity; condition which investigators judge to represent significant hazards; MACE including Death, MI, target lesion and vessel revascularization, or stent thrombosis will be classified as SAE

**6.4.2. Classification of Adverse Events**

Adequate review, assessment, and monitoring of adverse events require that they be classified as to severity, expectedness, and potential relatedness to the study intervention.

**6.4.2.1. Severity**

**Mild:** Awareness of signs or symptoms, but easily tolerated and are of minor irritant type causing no loss of time from normal activities. Symptoms do not require therapy or a medical evaluation; signs and symptoms are transient.

**Moderate:** Events introduce a low level of inconvenience or concern to the participant and may interfere with daily activities, but are usually improved by simple therapeutic measures; moderate experiences may cause some interference with functioning

**Severe:** Events interrupt the participant’s normal daily activities and generally require systemic drug therapy or other treatment; they are usually incapacitating

**6.4.2.2. Expectedness**

**Unexpected:** Nature or severity of the event is not consistent with information about the condition under study or intervention in the protocol, consent form, product brochure, or investigator brochure.

**Expected:** Event is known to be associated with the intervention or condition under study.

**6.4.2.3. Relatedness**

**Definitely Related:** The adverse event is clearly related to the investigational agent/procedure – i.e. an event that follows a reasonable temporal sequence from administration of the study intervention, follows a known or expected response pattern to the suspected intervention, that is confirmed by improvement on stopping and reappearance of the event on repeated exposure and that could not be reasonably explained by the known characteristics of the subject’s clinical state.

**Possibly Related:** An adverse event that follows a reasonable temporal sequence from administration of the study intervention follows a known or expected response pattern to the suspected intervention, but that could readily have been produced by a number of other factors.

**Not Related:** The adverse event is clearly not related to the investigational agent/procedure. i.e. another cause of the event is most plausible; and/or a clinically plausible temporal sequence is inconsistent with the onset of the event and the study intervention and/or a causal relationship is considered biologically implausible.

**6.4.3. Reporting Process**

AEs and/or laboratory abnormalities must be reported. All AEs experienced by the participant during the time frame specified in the protocol (e.g., from the time study drug administration through the end of the study) are to be reported, as outlined in the protocol.

Serious Adverse Event Reporting: All SAEs require expedited reporting by the PI to DSMB within 24 hours of the event being reported to the investigator. The expedited report should be followed by detailed, written SAE report as soon as possible. Follow up information may be required and asked for by the independent safety monitoring body directly.

Unanticipated Problems (UP): UPs require expedited reporting by the PI to DSMB within 48 hours of the event being reported to the investigator unless they are also SAEs. UP reporting procedures must include a corrective plan and measures to prevent reoccurrence. It is recommended that such events be reported within 48 hours to NIA unless they are also SAEs. Follow up information may be required and asked for by the independent safety monitoring body directly.

**7. Study Definition**

**@ Cardiovascular Death**

Defined as 1) due to myocardial infarction, cardiac perforation or tamponade, arrhythmia, heart failure, aortic cause and stroke (including ischemic and hemorrhagic) within 30 days of the procedure; 2) related to the procedure; 3) due to a complication of the procedure; 4) any death in which a cardiac cause cannot be excluded, as adjudicated by blinded clinical events committee.

**@ Major cardiovascular event**

Defined as coronary or peripheral revascularization, or hospitalization for cardiovascular events.

**@ Coronary or peripheral revascularization**

Defined as an endovascular and surgical revascularization of the coronary artery, carotid artery, or lower extremity artery. The need for coronary revascularization is based on typical symptoms and signs of electrocardiographic changes, exercise or pharmacological stress study evidence for inducible myocardial ischemia, angiographic evidence for new or worsening coronary artery disease and/or intracoronary thrombus. The need for lower extremity artery revascularization is based on the presence of intermittent claudication, rest pain, and /or ischemic ulceration in addition to stenosis or total occlusions. The need for carotid artery revascularization is based on the presence of symptoms or carotid artery stenosis greater than 80% in patients without symptoms at a discretions of neurologist.

**@ Hospitalization for cardiovascular events**

Defined as a hospitalization for ischemic heart disease, heart failure, or peripheral artery disease.

**@ Hospitalization for ischemic heart disease**

Defined as a hospitalization due to the need for coronary revascularization based on typical symptoms and signs of electrocardiographic changes, exercise or pharmacological stress study evidence for inducible myocardial ischemia, angiographic evidence for new or worsening coronary artery disease and/or intracoronary thrombus, or a hospitalization requiring at least an overnight stay due to substantial worsening of ischemic symptoms and signs (electrocardiographic, echocardiographic or biomarker changes).

**@ Hospitalization for Heart Failure**

Defined as an event that meets ALL of the following criteria:

1) The patient is admitted to the hospital with a primary diagnosis of HF

2) The patient’s length-of-stay in hospital extends for at least 24 hours

3) The patient exhibits documented new or worsening symptoms due to HF on presentation, including at least ONE of the following:

a. Dyspnea (dyspnea with exertion, dyspnea at rest, orthopnea, paroxysmal nocturnal dyspnea)

b. Decreased exercise tolerance

c. Fatigue

d. Other symptoms of worsened end-organ perfusion or volume overload (must be specified and described by the protocol)

4) The patient has objective evidence of new or worsening HF, consisting of at least TWO physical examination findings OR one physical examination finding and at least ONE laboratory criterion, including:

a. Physical examination findings considered to be due to heart failure, including new or worsened:

i. Peripheral edema

ii. Increasing abdominal distention or ascites (in the absence of primary hepatic disease)

iii. Pulmonary rales/crackles/crepitations

iv. Increased jugular venous pressure and/or hepatojugular reflux

v. S3 gallop

vi. Clinically significant or rapid weight gain thought to be related to fluid retention

b. Laboratory evidence of new or worsening HF, if obtained within 24 hours of presentation, including:

i. Increased B-type natriuretic peptide (BNP)/ N-terminal pro-BNP (NT-proBNP) concentrations consistent with decompensation of heart failure (such as BNP > 500 pg/mL or NT-proBNP > 2,000 pg/mL). In patients with chronically elevated natriuretic peptides, a significant increase should be noted above baseline.

ii. Radiological evidence of pulmonary congestion

iii. Non-invasive diagnostic evidence of clinically significant elevated left- or right-sided ventricular filling pressure or low cardiac output. For example, echocardiographic criteria could include: E/e’ > 15 or D-dominant pulmonary venous inflow pattern, plethoric inferior vena cava with minimal collapse on inspiration, or decreased left ventricular outflow tract (LVOT) minute stroke distance (time velocity integral (TVI))

OR

iv. Invasive diagnostic evidence with right heart catheterization showing a pulmonary capillary wedge pressure (pulmonary artery occlusion pressure) ≥18 mmHg, central venous pressure ≥12 mmHg, or a cardiac index < 2.2 L/min/m^2^

Note: All results from diagnostic tests should be reported, if available, even if they do not meet the above criteria, because they provide important information for the adjudication of these events.

5) The patient receives initiation or intensification of treatment specifically for HF, including at least ONE of the following:

a. Augmentation in oral diuretic therapy

b. Intravenous diuretic or vasoactive agent (e.g., inotrope, vasopressor, or vasodilator)

c. Mechanical or surgical intervention, including:

i. Mechanical circulatory support (e.g., intra-aortic balloon pump, ventricular assist device, extracorporeal membrane oxygenation, total artificial heart)

ii. Mechanical fluid removal (e.g., ultrafiltration, hemofiltration, dialysis)

**@ Hospitalization for peripheral artery disease**

Defined as a hospitalization due to revascularization and/or major or minor amputations.

**@ Nonfatal stroke**

Defined as a sudden focal neurologic deficit of presumed cerebrovascular etiology that persisted beyond 24 hours and is not due to another identifiable cause. An event matching this definition but lasting less than 24 hours is considered to be a transient ischemic attack. Brain imaging (computed tomography or magnetic resonance imaging) is recommended for all suspected strokes.

**@ New-onset diabetes mellitus**

Defined as initiating antidiabetic medication during study period, or in-study fasting plasma glucose >125 mg/dL.

**@ Muscle-relate adverse events**

Muscle-related adverse events include myalgia, myopathy, myositis, myonecrosis, and rhabdomyolysis with or without acute kidney injury according to the the 2014 National Lipid Association Statin Muscle Safety Task Force.

- Myalgia: A symptom of muscle-discomfort, including muscle aches, soreness, stiffness, tenderness, or cramps with or soon after exercise, with a normal creatine kinase (CK) level. Myalgia symptoms can be described as similar to what would be experienced with a viral syndrome such as influenza.

- Myopathy: Muscle weakness (not due to pain), with or without an elevation in CK level.

- Myonecrosis: Elevation in muscle enzymes compared with either baseline CK levels (while not on statin therapy) or the upper limit of normal: classified with mild (3-10x ULN), moderate (10-50x ULN), severe (>50x ULN).

**@ Aminotransferase elevation**

Increase from baseline and > 3 x ULN (upper limit of normal).

**@ Creatinine kinase elevation**

Definition of Creatine kinase elevation was Creatine kinase increase from baseline and > 5 x ULN (upper limit of normal).

**8. References**

1. Go AS, Mozaffarian D, Roger VL, Benjamin EJ, Berry JD, Borden WB, et al. Heart Disease and Stroke Statistics—2013 Update: A Report From the American Heart Association. Circulation. 2013;127(1):e6-e245.

2. Berliner JA, Navab M, Fogelman AM, Frank JS, Demer LL, Edwards PA, et al. Atherosclerosis: Basic Mechanisms: Oxidation, Inflammation, and Genetics. Circulation. 1995;91(9):2488-96.

3. Moreno PR, Fuster V. The year in atherothrombosis. Journal of the American College of Cardiology. 2004;44(11):2099-110.

4. Randomised trial of cholesterol lowering in 4444 patients with coronary heart disease: the Scandinavian Simvastatin Survival Study (4S). Lancet. 1994;344(8934):1383-9.

5. Sacks FM, Pfeffer MA, Moye LA, Rouleau JL, Rutherford JD, Cole TG, et al. The effect of pravastatin on coronary events after myocardial infarction in patients with average cholesterol levels. Cholesterol and Recurrent Events Trial investigators. The New England journal of medicine. 1996;335(14):1001-9.

6. Shepherd J, Cobbe SM, Ford I, Isles CG, Lorimer AR, MacFarlane PW, et al. Prevention of coronary heart disease with pravastatin in men with hypercholesterolemia. West of Scotland Coronary Prevention Study Group. The New England journal of medicine. 1995;333(20):1301-7.

7. Law MR, Wald NJ, Thompson SG. By how much and how quickly does reduction in serum cholesterol concentration lower risk of ischaemic heart disease?1994 1994-02-05 08:00:00. 367-72 p.

8. Baigent C, Keech A, Kearney Pa, Blackwell L, Buck G, Pollicino C, et al. Cholesterol Treatment Trialists’(CTT) Collaborators. Efficacy and safety of cholesterol-lowering treatment: prospective meta-analysis of data from 90,056 participants in 14 randomised trials of statins. Lancet. 2005;366(9493):1267-78.

9. LaRosa JC, Grundy SM, Waters DD, Shear C, Barter P, Fruchart J-C, et al. Intensive Lipid Lowering with Atorvastatin in Patients with Stable Coronary Disease. New England Journal of Medicine. 2005;352(14):1425-35.

10. Boekholdt SM, Hovingh GK, Mora S, Arsenault BJ, Amarenco P, Pedersen TR, et al. Very low levels of atherogenic lipoproteins and the risk for cardiovascular events: a meta-analysis of statin trials. Journal of the American College of Cardiology. 2014;64(5):485-94.

11. Grundy SM, Cleeman JI, Merz CNB, Brewer HB, Clark LT, Hunninghake DB, et al. Implications of Recent Clinical Trials for the National Cholesterol Education Program Adult Treatment Panel III Guidelines. Circulation. 2004;110(2):227-39.

12. European Association for Cardiovascular P, Rehabilitation, Reiner Z, Catapano AL, De Backer G, Graham I, et al. ESC/EAS Guidelines for the management of dyslipidaemias: the Task Force for the management of dyslipidaemias of the European Society of Cardiology (ESC) and the European Atherosclerosis Society (EAS). European heart journal. 2011;32(14):1769-818.

13. Stone NJ, Robinson JG, Lichtenstein AH, Bairey Merz CN, Blum CB, Eckel RH, et al. 2013 ACC/AHA Guideline on the Treatment of Blood Cholesterol to Reduce Atherosclerotic Cardiovascular Risk in Adults. Journal of the American College of Cardiology. 2014;63(25):2889-934.

14. Morris PB, Ballantyne CM, Birtcher KK, Dunn SP, Urbina EM. Review of clinical practice guidelines for the management of LDL-related risk. Journal of the American College of Cardiology. 2014;64(2):196-206.

15. Nakamura H, Arakawa K, Itakura H, Kitabatake A, Goto Y, Toyota T, et al. Primary prevention of cardiovascular disease with pravastatin in Japan (MEGA Study): a prospective randomised controlled trial. The Lancet.368(9542):1155-63.

16. Silva M, Matthews ML, Jarvis C, Nolan NM, Belliveau P, Malloy M, et al. Meta-analysis of drug-induced adverse events associated with intensive-dose statin therapy. Clinical Therapeutics.29(2):253-60.

17. Smith SC, Jr., Grundy SM. 2013 ACC/AHA guideline recommends fixed-dose strategies instead of targeted goals to lower blood cholesterol. Journal of the American College of Cardiology. 2014;64(6):601-12.

18. Gibson CM, Pride YB, Hochberg CP, Sloan S, Sabatine MS, Cannon CP. Effect of Intensive Statin Therapy on Clinical Outcomes Among Patients Undergoing Percutaneous Coronary Intervention for Acute Coronary SyndromePCI-PROVE IT: A PROVE IT–TIMI 22 (Pravastatin or Atorvastatin Evaluation and Infection Therapy–Thrombolysis In Myocardial Infarction 22) Substudy. Journal of the American College of Cardiology. 2009;54(24):2290-5.

19. Silva MA, Swanson AC, Gandhi PJ, Tataronis GR. Statin-related adverse events: A meta-analysis. Clinical Therapeutics. 2006;28(1):26-35.

20. Cannon CP, Blazing MA, Giugliano RP, McCagg A, White JA, Theroux P, et al. Ezetimibe added to statin therapy after acute coronary syndromes. New England Journal of Medicine. 2015;372(25):2387-97.

21. Virani SS, Woodard LD, Akeroyd JM, Ramsey DJ, Ballantyne CM, Petersen LA. Is High‐Intensity Statin Therapy Associated With Lower Statin Adherence Compared With Low‐to Moderate‐Intensity Statin Therapy? Implications of the 2013 American College of Cardiology/American Heart Association Cholesterol Management Guidelines. Clinical cardiology. 2014;37(11):653-9.

22. Foody JM, Toth PP, Tomassini JE, Sajjan S, Ramey DR, Neff D, et al. Changes in LDL-C levels and goal attainment associated with addition of ezetimibe to simvastatin, atorvastatin, or rosuvastatin compared with titrating statin monotherapy. Vascular health and risk management. 2013;9:719-27.

23. Toth PP, Ballantyne CM, Davidson MH, Tomassini JE, Ramey DR, Neff D, et al. Changes in prescription patterns before and after reporting of the Ezetimibe and Simvastatin in Hypercholesterolemia Enhances Atherosclerosis Regression trial (ENHANCE) results and expected effects on low-density lipoprotein-cholesterol reduction. Journal of clinical lipidology. 2012;6(2):180-91.

24. Baigent C, Blackwell L, Emberson J, Holland L, Reith C, Bhala N, et al. Efficacy and safety of more intensive lowering of LDL cholesterol: a meta-analysis of data from 170,000 participants in 26 randomised trials. Lancet. 2010;376(9753):1670-81.

25. Ray KK, Kastelein JJ, Boekholdt SM, Nicholls SJ, Khaw KT, Ballantyne CM, et al. The ACC/AHA 2013 guideline on the treatment of blood cholesterol to reduce atherosclerotic cardiovascular disease risk in adults: the good the bad and the uncertain: a comparison with ESC/EAS guidelines for the management of dyslipidaemias 2011. Eur Heart J. 2014;35(15):960-8.

26. Tonelli M, Muntner P, Lloyd A, Manns BJ, Klarenbach S, Pannu N, et al. Risk of coronary events in people with chronic kidney disease compared with those with diabetes: a population-level cohort study. Lancet (London, England). 2012;380(9844):807-14.

27. Tonelli M, Wanner C. Lipid management in chronic kidney disease: synopsis of the Kidney Disease: Improving Global Outcomes 2013 clinical practice guideline. Annals of internal medicine. 2014;160(3):182.

28. Arai H, Sasaki J, Teramoto T. Comment on the new guidelines in USA by the JAS guidelines committee. Journal of atherosclerosis and thrombosis. 2014;21(2):79-81.

29. Rosenson RS, Baker SK, Jacobson TA, Kopecky SL, Parker BA, The National Lipid Association's Muscle Safety Expert P. An assessment by the Statin Muscle Safety Task Force: 2014 update. Journal of clinical lipidology. 2014;8(3 Suppl):S58-71.

**Summary of Changes**

**Protocol Amendment Date: 01 Jun 2021**

**Modification 1:** Due to the retirement of Professor Yangsoo Jang from Severance Hospital, Yonsei University College of Medicine, the principal investigator was change to Professor Myeong-Ki Hong.

**Original:**

Principle Investigator:

**Yangsoo Jang, MD, PhD**

**Revised to:**

Principle Investigator:

**Myeong-Ki Hong, MD, PhD**

**Protocol Amendment Date: 23 Mar 2022**

**Modification 1:** A typo was found regarding the number of participating centers in the trial.

**Original:**

**24** centers including Severance Hospital

50-1 Yonsei-ro, Seodaemun-gu, Shinchondong, Seoul, South Korea 03722

**Revised to:**

**26** centers including Severance Hospital

50-1 Yonsei-ro, Seodaemun-gu, Shinchondong, Seoul, South Korea 03722
